# An Aggressive Cuckoo Search Algorithm for Optimum Power Allocation in a CDMA-Based Cellular Network

**DOI:** 10.1155/2022/5443160

**Published:** 2022-08-30

**Authors:** Shawn Muthomi Mwitia, Davies Rene Segera

**Affiliations:** Department of Electrical and Information Engineering, University of Nairobi, Nairobi 30197, Kenya

## Abstract

This paper proposes an aggressive cuckoo search algorithm for optimum power allocation in a CDMA-based cellular network. To make the cuckoo search algorithm aggressive, adaptive parameters are used to vary the step size and probability of discovery. Furthermore, the Lévy flight is replaced with the Beta distribution to further improve the performance of the algorithm. To prove that the proposed algorithm is superior, the algorithm is tested on 23 benchmark test functions and its results are compared with those of 10 other standard optimization algorithms and 4 other advanced optimization algorithms. The performance of the proposed algorithm is proved via the statistical analysis of the results using the Wilcoxon rank-sum test. The proposed algorithm is then utilized in determining the optimal uplink power for multiple users in a CDMA-based cellular network in three different scenarios through Rician fading channels. The resultant allocated power should ensure that each mobile station meets its predetermined signal-to-interference-and-noise ratio while utilizing the least amount of power.

## 1. Introduction

In the field of engineering, many design problems involve the determination of the best solution from multiple possibilities containing different parameters and conditions under complex constraints. There are a wide variety of constraints that an engineer would have to take into account, such as the range of material properties or even the load capacity of a machine [1]. This brings about the need for optimization in engineering. The goal of optimization is to determine either the minimum or maximum point of the function being solved [2]. Optimization is applied in other aspects of engineering other than design, such as in information systems to create deployment strategies for cloud computing services or in electrical engineering to forecast energy consumption [3].

Many types of optimization algorithms exist, such as bracketing algorithms, first-order algorithms, and direct algorithms. Of these algorithms, a recent trend has seen the use of evolutionary algorithms, such as genetic algorithms (GA) and genetic programming, and swarm-based algorithms increasing. Examples of swarm-based algorithms include ant colony optimization, particle swarm optimization (PSO), firefly algorithm (FA), grey wolf optimization algorithm (GWO), and the cuckoo search algorithm [4]. These algorithms are inspired by nature. The flexible, efficient, and highly adaptable nature of these algorithms is the reason behind their increased use in optimization. Likewise, these algorithms can easily be implemented in a large array of programming languages, thus enabling their use in a wide variety of cases [5].

Evolutionary algorithms are a collective of algorithms that adapt their population with each generation by modifying their potential solutions by randomly discarding poor solutions and only permitting the fit solutions to move on to the next generation. Although evolutionary algorithms can solve unstructured problems and do not require differentiability of their objective functions, they are typically avoided as they are not suitable for solving large-scale problems due to their intensive computational needs, thus leading to the preference for swarm-based algorithms [6].

In the development of these nature-inspired optimization algorithms, two factors are taken into account in their evaluation: exploration and exploitation. Exploration refers to the searching of the global search space, while exploitation refers to searching of the local search space. These two factors need to be balanced in the development of the optimization algorithm [7, 8]. Swarm-based algorithms consist of two phases, namely, the variation and selection phases. The variation phase searches the search space and the selection phase exploits the identified search space based on previous experiences. These two phases maintain a balance between exploration and exploitation thus leading swarm-based algorithms to be the preferred optimization algorithm [6].

Among swarm-based algorithms, the cuckoo search algorithm is determined to be efficient. This is due to the two search capabilities of the cuckoo search algorithm: the local and global search, being controlled by a switching probability. This enables the algorithm to more efficiently search the global space compared to other swarm-based algorithms such as PSO. Furthermore, the PSO algorithm has been deduced to converge quickly to the current best solution but not always to the global best solution [9, 10].

Due to the above-stated reasons, the cuckoo search algorithm is one of the most preferred optimization algorithms over other algorithms such as PSO and GA. Although the cuckoo search algorithm is viewed as a highly efficient optimization algorithm, it is not perfect. The algorithm can easily converge to the local optimum solution, and the algorithm generally has a slow rate of convergence [11, 12].

The purpose of this study is to mitigate the above problems mentioned by proposing an aggressive cuckoo search algorithm (ACSA). The aggressive nature of the algorithm comes about by using an adaptive step size and probability of discovery whose values would change with each iteration of the algorithm. Likewise, to further improve the performance of the ACSA, the Lévy distribution is replaced with a beta distribution. The ACSA will be used in determining the optimal uplink power for multiple users in a CDMA-based cellular network.

## 2. Literature Review

### 2.1. Improvements to the Standard Cuckoo Search Algorithm

Although the cuckoo search algorithm is a widely used optimization algorithm due to its simple implementation, it suffers from some downfalls. In [13], the authors expressed some of the problems that the standard cuckoo search algorithm suffers from, such as the reduced global exploration ability due to the algorithm's random initialization of its population. Furthermore, the Lévy flight step size scaling factor is constant, thus this parameter needs to be tuned for a particular problem, and this increases the difficulty of using the algorithm.

Due to this issue, many researchers have developed improvements to the standard cuckoo search algorithm that mitigate the above problem. In [14], Meng et al. described that there are four main methods to improve the standard cuckoo search algorithm. The first method is to use an adaptive parameter in the algorithm to enable the parameters of the algorithm to dynamically change. The second method is by replacing the Lévy flight method with other better search strategies such as the random long-distance search strategy or the stochastic short-distance strategy. The third method is the hybridization of the cuckoo search algorithm with other optimization algorithms. The last method is improving the initial solutions.

### 2.2. Review of Cuckoo Search Algorithms with Adaptive Parameters

In [15], Zhang and Chen noticed that the step length of the Lévy flight was constant and thus proposed making the Lévy flight step size a value that reduced with increasing generation. He concluded that with the improvements, the new cuckoo search algorithm had a faster convergence speed and higher precision than the standard cuckoo search algorithm. A self-adaptive cuckoo search algorithm was proposed in [16]. In this proposal, the adaptive nature of the algorithm was employed through a linear population reduction. The linear population reduction reduced the total number of function evaluations required and thus enabled better exploration toward the end of the iterations. The self-adaptive cuckoo search algorithm achieved better results than the standard cuckoo search algorithm and the self-adaptive differential evolution algorithm.

In [17], the authors proposed an adaptive cuckoo search algorithm for searching for optimal network configuration and distributed generation allocation. The cuckoo search algorithm was made adaptive by using graph theory to reduce the number of infeasible individuals. In most scenarios, this adaptive algorithm outperforms a firework optimization algorithm and harmony search algorithm in determining the network configuration that minimized the active power loss and enhanced the voltage stability index of the power distribution system. Furthermore, an improved cuckoo search algorithm was proposed in [18]. This algorithm was made adaptive by using a convergence improvement strategy, and it was tested against the standard cuckoo search algorithm and it outperformed it in 32 out of 34 test cases. This enabled the author to optimally determine the roots of nonlinear equation systems.

### 2.3. Review of the Cuckoo Search Algorithms with Replaced Lévy Flight

In [19], the authors present a hybrid many-objective cuckoo search algorithm that uses both Lévy and exponential distribution. The authors compared their algorithm with other variations of the cuckoo search algorithm that used different combinations of the Lévy distribution, Cauchy distribution, and exponential distribution. In their analysis, they discovered that the Lévy and exponential distribution combination had the best performance of the other combinations and outperformed the Multi-objective Evolutionary Algorithm Based on Decomposition (MOEA/D) in the popular WFG suite. In [20], an enhanced fractional-order cuckoo search optimizer using heavy-tailed distributions is used to classify COVID-19 X-ray images. The authors utilized the fractional-order cuckoo search (FO-CS) algorithm with a wide variety of heavy-tailed distributions, i.e., the Lévy distribution, Mittag-Leffler distribution, Pareto distribution, Cauchy distribution, and Weibull distribution. The FO-CS variants were also tested against the following algorithms: Harris hawks optimization (HHO), Henry gas solubility optimization (HGSO), Genetic Algorithm (GA), Swarm Algorithm (SSA), Whale optimization algorithm (WOA), and the Grey Wolf Optimizer (GWO). The authors concluded that the use of heavy-tailed distribution can be used to prevent local trapping by the algorithm and escape from the non-prominent regions of the search space. The authors also concluded that the FO-CS variant with Weibull distribution generally outperformed the other FO-CS variants in their various tests such as in feature selection and in their mean fitness function mean values.

### 2.4. Review of the Hybridization of the Cuckoo Search Algorithm with Other Metaheuristic Algorithms

In [21], the authors describe that in population-based search algorithms, the parameter settings of the algorithm and the present population diversity can have a great impact on the performance of the algorithm. The higher the population diversity of the algorithm, the better the exploration by the search algorithm. This brings about the need for the hybridization of population-based search algorithms. In [22], a proposal was made for hybridizing the harmony search algorithm with the cuckoo search algorithm. The pitch adjustment operation in the harmony search algorithm was added to the cuckoo search algorithm as a mutation operator. It was concluded that the hybridized cuckoo search algorithm avoided premature convergence caused by getting trapped in local optimum regions and thus outperformed the standard cuckoo search algorithm.

In [23], a hybridization of the cuckoo search algorithm and particle swarm optimization was proposed. The hybrid algorithm has an enhanced diversity of optimal solutions and convergence solutions. This resulted in the algorithm outperforming the standard cuckoo search algorithm. On top of that, a hybrid grey wolf optimizer and cuckoo search algorithm were proposed in [24] to be used in the extraction of parameters of solar photovoltaic models. The hybridization of the two algorithms aimed to balance global exploration and local exploitation. The hybrid algorithm was applied to solve ten global optimization problems with different characteristics and to four solar photovoltaic models for parameter extraction, and it was concluded that the hybrid algorithm had better robustness for parameter extraction and it had faster convergence speed than other algorithms such as the standard grey wolf optimizer, the improved grey optimizer, and even the whale optimization algorithm.

## 3. Standard Cuckoo Search Algorithm

### 3.1. Inspiration for the Cuckoo Search Algorithm

The Standard Cuckoo Search algorithm (SCSA) was developed by Yang and Deb [1, 9]. The algorithm is based on the brood parasitism nature of some species of cuckoo bird. The cuckoo bird lays its eggs in the nests of other host birds and thus its offspring would depend on the host bird for food and survival [25]. There is a probability that the host bird might notice the cuckoo bird's egg, and if the host bird does notice the egg, it may either remove the cuckoo bird's egg or abandon the nest entirely and build a new nest in a different place. To reduce the probability of discovery, the cuckoo bird lays eggs that mimic the host egg's color and pattern.

### 3.2. Random Walk and Lévy's Flight

In nature, animals search for food in a random or quasi-random manner in which the next move is based on the current location or state and transition probability to the next location. The direction chosen by the animal depends on a probability that can be modeled mathematically. To mimic this random movement Lévy's flight is employed, which derives its step length from the heavy-tailed Lévy distribution [26].

(1) $$\begin{array}{c} \text{Lévy}\left(\beta \right)=\frac{\phi *\mu }{{\left|v\right|}^{\left(1/\beta \right)}},\\ \phi ={\left(\frac{\Gamma \left(1+\beta \right)*\sin\left(\pi *\beta /2\right)}{\Gamma \left(\left(\left(1+\beta \right)/2\right)*\beta *2^{\left(\beta -1/2\right)}\right)}\right)}^{1/\beta }. \end{array}$$

The values *μ* and *v* are random numbers drawn from a normal distribution with a mean of 0 and a standard deviation of 1, and Γ is the gamma function. The Lévy exponent *β* is usually held constant throughout the operation of the algorithm.

### 3.3. Standard Cuckoo Search Algorithm

In the cuckoo search algorithm, each cuckoo egg represents a new solution while the host bird's eggs represent a new candidate solution [27]. To implement the cuckoo search algorithm, three idealized rules need to be taken into account [28]:

- Each cuckoo bird lays one egg and places it in a randomly chosen nest.
- The best nests with the highest quality eggs will carry over to the next generation.
- The number of available host nests is fixed and the host bird can discover the cuckoo bird's egg with a probability of discovery *Pa* ∈ [0,1]. In this situation, the host bird can either remove the alien cuckoo egg or abandon the nest entirely and build a new nest in a new location.

Based on these three rules, the pseudo-code of the standard cuckoo search algorithm can be summarized as shown in Algorithm 1.

As discussed earlier, optimization algorithms take into account two main phases: exploration and exploitation, and these two phases need are balanced in Algorithm 1 by the probability of discovery *P*_*a*_=0.25.

For nest *i*, its next generation is derived by the following equation:

(2) $$\begin{array}{c} x_{i}^{t+1}=x_{i}^{t}+\left(\alpha ⊕\text{Levy}\left(\beta \right)\right), \end{array}$$

where *x*_*i*_^*t*^ is the current generation of nest *i*, while *x*_*i*_^*t*+1^ is the new nest generated by Lévy flight. The product ⊕ means entry-wise multiplication. *α* is the step size and it must meet the condition *α* > 0.

## 4. Proposed Aggressive Cuckoo Search Algorithm

In this section, a new aggressive cuckoo search algorithm is proposed based on three modifications made to the standard cuckoo search algorithm.

### 4.1. Adaptive Step Size

In the standard cuckoo search algorithm, a constant step size is employed in the Lévy flight. A common observation is that when the step size is large, the performance of the algorithm is slow but the algorithm does reach the global optimum after a large number of iterations. If the step size is small, the algorithm has a high chance of converging to a local optimum. Due to this problem, an adaptive step size is proposed.

During the initialization of the population, a uniform distribution is employed, thus enabling a diverse distribution of the population throughout the entire search space. Therefore, a smaller step size can be employed in the initial iterations to enable each search agent efficiently search for the minimum in their diverse locations in the search space. However, as the iterations increase, the search agents may be trapped in a local minimum, thus warranting the need for a larger step size to enable the search agents to move out of their local minimum. Therefore, increasing the step size would enable better faster convergence of the cuckoo search algorithm compared to a constant step size.

Eber Moll's model of a transistor is emulated to get an equation for step size *α*.

(3) $$\begin{array}{c} \text{Is}=\text{Ies}\left(e^{\left(V_{BE}/V_{T}\right)}-1\right). \end{array}$$

This produces the following equation for step size *α*:

(4) $$\begin{array}{c} \text{Normalising Equation}=\frac{1}{e^{\left(\text{Maximum Iterations}-1/\text{Maximum Iterations}\right)}-1}, \end{array}$$

(5) $$\begin{array}{c} \text{factor}=\text{Normalizing Equation}*\left(e^{\left(\text{Iteration}/\text{Maximum Iterations}\right)}-1\right), \end{array}$$

(6) $$\begin{array}{c} \alpha_{\text{adaptive}}=\alpha_{\min}+\text{factor}*\left(\alpha_{\max}-\alpha_{\min}\right). \end{array}$$

**Figure 1:**
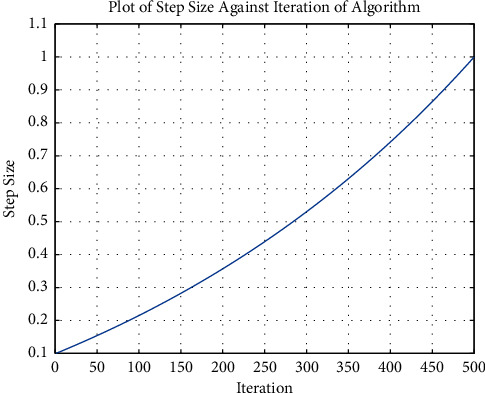
Plot showing the step size of the algorithm against the increasing number of iterations.

The values of *α*_min_ and *α*_max_ are predetermined before the execution of the algorithm.  Figure 1 shows the adapting nature of the algorithm as the iterations increase to a maximum of 500:

### 4.2. Adaptive Probability of Discovery

The probability of discovery controls the two search capabilities of the cuckoo search algorithm: local search (exploitation) and global search (exploration). In SCSA, the probability of discovery *Pa* is kept constant at 0.25. This means that the algorithm focuses on local search 25% of the time and focuses on the global search 75% of the time. Therefore, by increasing the probability of discovery from a minimum to the maximum predetermined value, the local search of the algorithm can be increased to coincide with the increasing local search caused by the adaptive step size.

The probability of discovery is made adaptive by making it vary linearly with respect to the iteration of the algorithm.

(7) $$\begin{array}{c} \text{Gradient}=\left(Pa_{\max}+Pa_{\min}\right)*\left(\frac{\text{Maximum Iterations}}{\text{Maximum Iterations}-1}\right), \end{array}$$

(8) $$\begin{array}{c} Pa_{\text{adaptive}}=\left(\frac{\text{Gradient}*\text{Iteration}}{\text{Maximum Iterations}}\right)+Pa_{\min}. \end{array}$$

**Figure 2:**
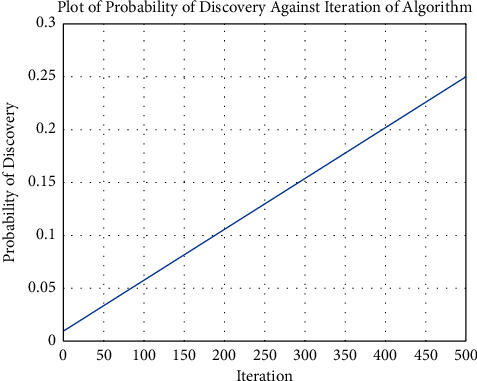
Plot showing the probability of discovery of the algorithm against the increasing number of iterations.

The values of *Pa*_min_ and *Pa*_max_ are predetermined before the execution of the algorithm.  Figure 2 shows the adapting nature of the probability of discovery as the iterations increase to a maximum of 500:

### 4.3. Replacement of Lévy Flight with Beta Distribution

The beta distribution is a continuous probability distribution defined in the interval [0, 1]. It is characterized by its widely varying shape due to the manipulation of its two parameters alpha and beta.

(9) $$\begin{array}{c} \text{Beta}\left(\alpha \text{,}\beta \right)=\frac{X_{i}^{\alpha -1}*{\left(1-X_{i}\right)}^{\beta -1}*\Gamma \left(\alpha +\beta \right)}{\Gamma \left(\alpha \right)*\Gamma \left(\beta \right)}, \end{array}$$

where Γ is the gamma function.

This widely varying shape is advantageous as it enables the creation of a distribution that adds to the increased convergence rate caused by the adaptive parameters. To further increase the convergence rate, a negatively skewed distribution is required, thus alpha must be greater than beta.

With the above three proposed changes to the cuckoo search algorithm, the generation of the next nest *i*, is derived from the following equation:

(10) $$\begin{array}{c} X_{i}^{t+1}=X_{i}^{t}+\left(\alpha_{\text{adaptive}}⊕r*\text{Beta}\left(\alpha ,\beta \right)\right), \end{array}$$

where *r*=[−1,0,1].

Based on the above-proposed changes, the pseudo-code of the aggressive cuckoo search algorithm is shown in Algorithm 2.

**Figure 3:**
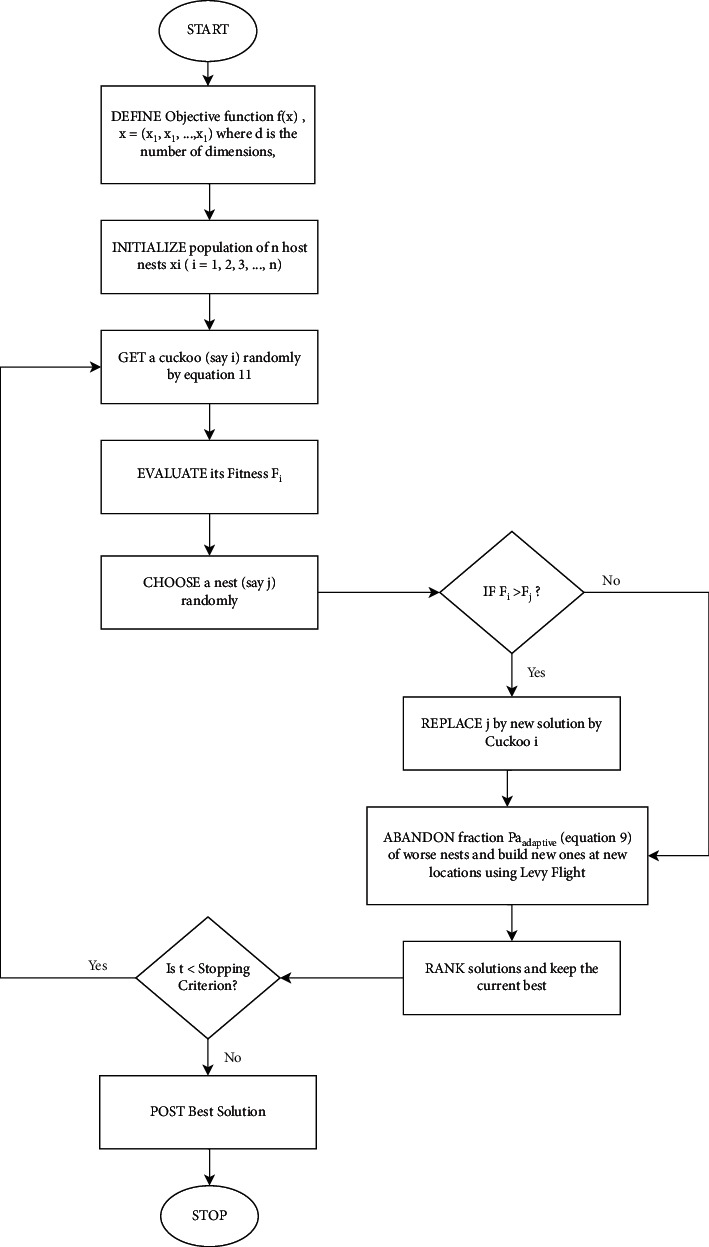
Flowchart representation of Algorithm 2.

A diagrammatic representation of Algorithm 2 is presented in Figure 3.

## 5. Implementation and Validation

In this section, the performance of the algorithms will be evaluated by using the algorithms to solve classical optimization benchmark functions utilized in the following optimization literature [29]. The benchmark functions are grouped into three categories: unimodal, multimodal, and fixed-dimension multimodal functions. The unimodal test functions verify the local search ability of the ACSA and are presented in Table 1, the multimodal benchmark functions are used to evaluate the global search capability and are presented in Table 2 and the fixed-dimension multimodal functions are used to evaluate the convergence accuracy and are presented in Table 3 [30].

### 5.1. Ablation Experiment on the Varying Step Size

**Figure 4:**
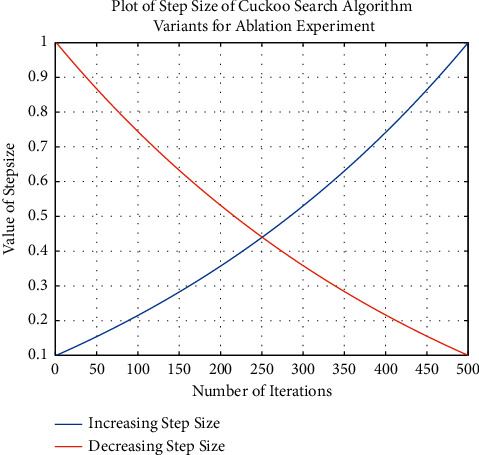
Plot of step size for cuckoo search algorithm variants for the ablation experiment.

In order to determine if the increasing step size of the algorithm increases the performance of the algorithm by a greater magnitude as compared to an algorithm with decreasing step size, a variant of the cuckoo search algorithm is implemented that only has the adaptive step size implemented in equation (6). In this implementation, the step size is increasing. Another variant of the cuckoo search algorithm is implemented that has an adaptive step size that decreases as the iterations of the algorithm increase. The varying step sizes of the two cuckoo search algorithm variants are shown in Figure 4.

The two cuckoo search algorithm variants are used to solve the 23 classical benchmark optimization functions presented in Tables 1–3. For each benchmark function, the algorithms were tested with a maximum number of 500 iterations and a population of 50 starting from randomly generated initial populations. Each algorithm was run 15 times independently for each benchmark function and the average of the obtained minimum values and their standard deviation have been recorded in Table 4. The following results were obtained by running the algorithm on an ASUS Intel Core i5-8250U @1.60 GHz laptop with 6.00 GB RAM.

Of the 23 benchmark functions, the cuckoo search variant with the increasing step size outperformed the other variant in nine functions. The variant with a decreasing step size outperformed in five functions, and the two variants obtained the same value in nine functions.

### 5.2. Evaluation Metrics

In this section, the ACSA is initially compared with ten other standard optimization algorithms to evaluate its performance. These algorithms are: Artificial Bee Colony (ABC) algorithm, Bat Algorithm (BA), Cultural Algorithm (CA), Differential Evolution (DE) algorithm, Firefly Algorithm (FA), Flower Pollination Algorithm (FPA), Genetic Algorithm (GA), Invasive Weed Optimization (IWO) algorithm, Particle Swarm Optimization (PSO) algorithm and the Standard Cuckoo Search algorithm (SCSA).

For each benchmark function, the algorithms were tested with a maximum number of 500 iterations and a population of 50 starting from randomly generated initial populations. Each algorithm was run 15 times independently for each benchmark function and the average of the obtained minimum values and their standard deviation have been recorded in Table 5. If multiple algorithms achieved the same mean, the algorithm with the lower standard deviation was chosen as the best performing algorithm.

From the results in Table 5, it can be seen that the ACSA outperformed the other algorithms or reached the same optimum value as some of the other algorithms in 18 of the 23 benchmark functions, with the ACSA only being outperformed in benchmark functions *F*5, *F*6, *F*8, *F*12, and *F*13. The ACSA always outperformed or reached the same optimum value as the SCSA in all 23 functions.

**Figure 5:**
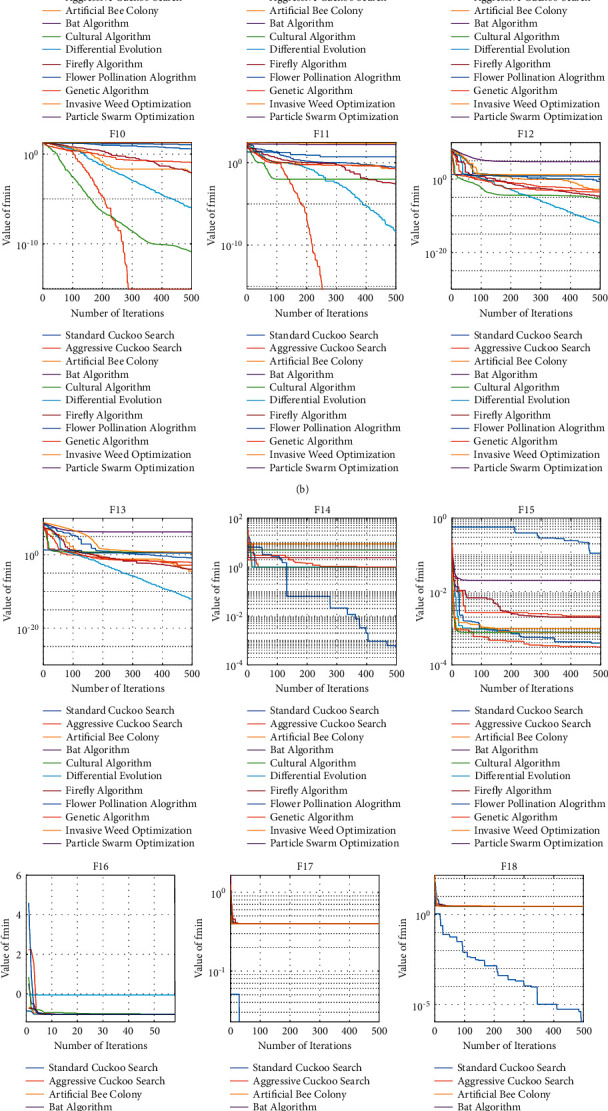
Comparison of the convergence curves of ACSA with the other optimization algorithms for all benchmark optimization functions.

The plot of each algorithm's convergence curve against each test function is shown in Figure 5.

From the convergence curves, it can be seen that the aggressive cuckoo search algorithm has a faster convergence rate compared to the other optimization algorithms, like in functions *F*1, *F*2, *F*3, *F*4, *F*9, *F*10, and *F*11.

To further test the performance of the ACSA, it is compared against the following advanced optimization algorithms: Fuzzy Self-Tuning Differential Evolution (FSTDE) algorithm [31], Ranking-based Adaptive Cuckoo Search (RACS) algorithm [32], Improved Real-Coded Genetic Algorithm (IRGA) [33], and Gaussian Quantum-behaved Particle Swarm Optimization (GQPSO) [34] algorithm. The FSTDE algorithm utilizes fuzzy logic to determine the parameters for each solution, the RACS uses a ranking-based crossover in its mutation strategy, the IRGA utilizes a directional crossover to improve the performance of a real-coded GA and the GQPSO uses a modified PSO algorithm that utilizes a mutation operator with a Gaussian probability distribution. For each benchmark function, the algorithms were tested with a maximum number of 500 iterations and a population of 50 starting from randomly generated initial populations. Each algorithm was run 15 times independently for each benchmark function and the average of the obtained minimum values and their standard deviation have been recorded in Table 6. If multiple algorithms achieved the same mean, the algorithm with the lower standard deviation was chosen as the best performing algorithm.

Of the 23 functions, the ACSA either outperformed or matched the performance of another algorithm in 13 functions. The ACSA was mainly outperformed in the unimodal functions by the GQPSO due to its superior local search capability. The GQPSO struggled in the multimodal functions, particularly in the fixed-dimension multimodal functions.

### 5.3. Timing Analysis

The execution time of each of the algorithms can be used to determine the performance of the aggressive cuckoo search algorithm. The average time of running each of the algorithms 15 times has been presented in Table 7.

From the results of Table 7, it can be seen that the PSO algorithm has the shortest runtime in 16 functions, i.e., in approximately 69.5% of all the benchmark functions. This is due to the algorithm having few parameters to tune, thus executing quickly [35].

The ACSA consistently had around the fifth or sixth shortest execution time among the 10 standard optimization algorithms. Although, this is due to the algorithm being compared to consistently fast algorithms such as the flower pollination algorithm which is characterized by being simple in its formulation and thus having a high computational performance [36].

The ACSA has a faster execution time compared to the SCSA in 17 of the 23 benchmark functions, with the SCSA mainly outperforming it in functions *F*14, *F*16, *F*17, *F*18, *F*19, and *F*23. On top of that, the SCSA was quicker in the fixed-dimension multimodal benchmark functions which had fewer dimensions, i.e., from 2–6, and was slower in the unimodal and multimodal benchmark functions which had 15 dimensions. Therefore, the modifications in the aggressive cuckoo search algorithm improved the execution time of the algorithm when dealing with objective functions with many dimensions but slowed down the algorithm when dealing with few dimensions. This improves the performance of the ACSA when dealing with complex objective functions that may have numerous dimensions for its variables.

**Figure 6:**
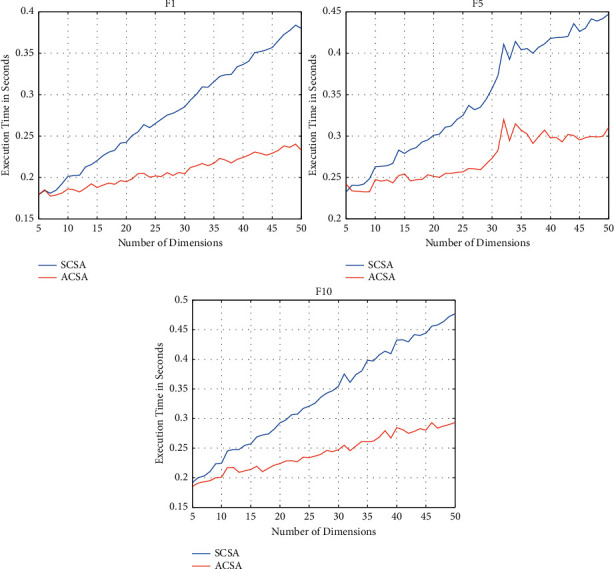
Plots of execution times of SCSA and ACSA against number of dimensions of benchmark functions.

To analyze the time complexity of the ACSA, the SCSA and ACSA were used to solve the optimization functions *F*1, *F*5, and *F*10 with varying dimensionality for each function. For each function, each algorithm solved the function 15 times for varying dimensionality starting from 5 to 50. The mean timings for each algorithm for each number of dimensions have been plotted in Figure 6.

From the three plots in Figure 6, it can be seen that the execution time of the SCSA increases by a greater rate with the increase in the number of dimensions of the objective function as compared to the ACSA. This is beneficial to the ACSA when solving problems with a large number of variables as the algorithm will iterate quicker and thus converge at the optimum solution quicker.

### 5.4. Statistical Analysis of Evaluation Metrics

In this section, the ACSA is compared to each of the 10 standard optimization algorithms in each of the 23 test functions using the Wilcoxon ranked sum test to determine if there is a significant difference between the two algorithms. The Wilcoxon ranked sum test was carried out with a 5% significance level. An *h*-value of 1 demonstrates that there is a significant difference between the two algorithms, whereas an *h*-value of 0 demonstrates the opposite. The statistical analysis results have been presented in Table 8. From Table 8, of the 230 comparisons done between the ACSA and the other optimization algorithms, 207 resulted in an *h*-value of 1. Therefore, 90% of all 230 comparisons had different results between the two algorithms.

## 6. Application of the Aggressive Cuckoo Search for Optimized Uplink Power Control in CDMA-Based Cellular Networks

CDMA networks enable multiple users to communicate via a single transmission channel by optimizing the use of the available bandwidth. The use of this single transmission channel leads to the problem of interference between multiple users, as the users transmit their data using the same frequency.

**Figure 7:**
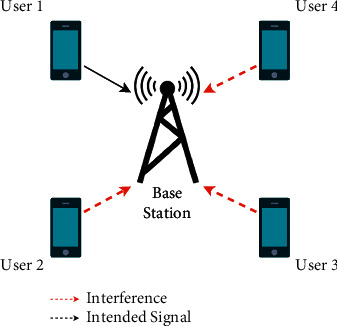
CDMA-based system with 4 users.

Furthermore, the near-far effect degrades the quality of the received signal at the base station. This is a phenomenon that arises when a mobile station near the base station transmits a signal that overpowers the signal from another mobile station that is farther from the base station [37]. The signal from the farther mobile station is weaker due to path loss as the signal has to travel a longer distance to reach the base station. On top of that, the signal may encounter objects such as trees and buildings in its path, which would cause scattering and diffraction of the signal.  Figure 7 shows the interference that multiple users would cause on the signal from one user.

Power control is needed in this system to ensure that a predetermined Quality of Service (QoS) is met while still ensuring the least amount of power is required by the transmitting Mobile Stations (MS). In this use case, the QoS requirement for each user is the Signal-to-Interference and Noise Ratio (SINR).

The SINR for the i^th^ user is determined as follows:

(11) $$\begin{array}{c} \gamma_{i}=\frac{H_{ii}p_{i}}{\sum_{j=1,j\neq 1}^{n}H_{ji}p_{j}+\;\sigma^{2}}. \end{array}$$

The *σ*^2^ is the additive white Gaussian noise (AWGN) and *p*_*i*_ is the power transmitted by user *i*. *H*_*ij*_ is the Rician fading component and channel gain from user *i* to *j*, and it takes into consideration the path loss and log-normal shadowing.

*H *_*ii*_ is the channel gain from user *i* to the base station and is written as follows [38, 39]:

(12) $$\begin{array}{c} H_{ii}=\;\bar{g}d_{ii}^{-\propto }10^{\Xi /10}, \end{array}$$

where $\bar{g}=0.97$, *d*_*ii*_ is the distance from user *i* to the base station, *α* is the path loss exponent, and Ξ is the Gaussian random variable that represents the shadowing.

The ACSA was tested in this electrical engineering design problem. The objective of the algorithm is to minimize the power consumption among all users while still meeting the required SINR value for each user.

The objective function is shown as follows:

(13) $$\begin{array}{c} \min\sum_{i=1}^{n}p_{i}, \end{array}$$

where *p*_*i*_ is the transmit power from the *i*^th^ MS and *n* is the total number of MS.

The constraints used in this optimization problem are as follows:

(14) $$\begin{array}{c} \frac{H_{ii}p_{i}}{\sum_{j=1,j\neq 1}^{n}H_{ji}p_{j}+\sigma^{2}}\geq \gamma_{i}^{th},\\ p_{i}\geq 0, \end{array}$$

where *γ*_*i*_ is the predetermined SINR value for MS *i*.

## 7. Results and Comparisons

The performance of the ACSA is tested in three scenarios. In the first scenario, there are five users, uniformly distributed in a 50 m × 50 m square geographical area. Each user is required to meet a target SINR of 3 dB. In the second scenario, there are five users, uniformly distributed in a 150 m × 150 m square geographical area, with each user required to meet a target SINR of 3 dB. In scenario three, there are six users uniformly distributed in a 200 m × 200 m square geographical area, with the required target SINR value being 3 dB. In all three scenarios, the base station is located at the center of the square geographical area.

The objective function was set as a penalty unconstrained function to take the constraints into account, with the penalty parameter being equal to 10^20^. The algorithms were run with a population size of 50 and for 1000 iterations. *α* was set as 4 for the simulation of an urban environment and noise was set as 0.0002 mW.

Due to the random positions, the mobile stations could take in each square geographical area, the algorithm was tested four times in each scenario, with one example being elaborated on further. Algorithms that returned values greater than their total allocated power did this due to the algorithm not being able to meet the SINR requirement for each user, thus a penalty value was added to the returned value. The SINR values have been determined by the power values derived by the algorithms. To extensively test the ACSA, the ACSA was compared against two sets of algorithms for each scenario, the standard and advanced optimization algorithms.

The total power for transmission together with the SINR values for each user have been presented in Tables9–14. The Tables 15–20 contain the extra test cases that algorithms were observed in for each scenario. 

### 7.1. Scenario 1

For scenario 1, the first test case's results are presented in Tables 9 and 10. From Table 9, the ACSA was able to allocate the least amount of power to each mobile station for signal transmission, while still ensuring that the required SINR value for uplink transmission was achieved by each mobile station. From Table 10, the RACS and IRGA outperformed the ACSA in ensuring that the least amount of power was utilized. The results of the remaining three test cases are presented in Tables 15 and 16. From Table 16, it can be seen that the RACS and IRGA algorithms still outperformed the ACSA.

### 7.2. Scenario 2

For scenario 2, the first test case's results are presented in Tables 11 and 12. Just as in scenario 1, in this scenario, as can be seen in Table 11, the ACSA was able to allocate the least amount of power to each mobile station for signal transmission among the standard optimization algorithms, while still ensuring that the required SINR value for uplink transmission was achieved by each mobile station. The results of the remaining three test cases are presented in Table 17. Among the advanced optimization algorithms, the ACSA was outperformed only by the RACS algorithm, and this can be seen from the results in Tables 12 and 18.

### 7.3. Scenario 3

For scenario 3, the first test case's results are presented in Tables 13 and 14. In this scenario, none of the algorithms could achieve the required 3 dB SINR value for each mobile station due to the upper bound set to prevent the algorithms from assigning power values of greater than 100 W. However, the ACSA managed to have the lowest returned value. This is because the returned value contains both the sum of the mobile stations' powers and the penalty value that was added due to not achieving the required SINR value. The magnitude of this penalty value is dependent on the difference between the achieved SINR value and the required value. This is the reason behind the ACSA's lowest returned value. It is because the achieved SINR values of its mobile stations were closest to the required SINR value of 3 dB.

This is the reason for choosing it as having allocated the best power values, even though its total power is greater than those of the CA, ABC, and RACS algorithms. It is because it managed to achieve closer SINR values to the required SINR value while still allocating low power to each mobile station. The results of the remaining test cases for both the standard and advanced optimization algorithms are presented in Tables 19 and 20.

As the difficulty in optimization increased from scenario 1 to 3, the ACSA's performance increased with respect to the other algorithms. For example from Tables 10, 12 and 14, it can be seen that in scenario 1 (Table 10) the RACS algorithm easily outperformed the ACSA. In scenario 2 (Table 12), the RACS algorithm still outperformed the ACSA but by a smaller margin. In scenario 3 (Table 14), the ACSA algorithm outperformed all the other algorithms. As the dimensionality and parameters increased, the ACSA's performance was slowed down by a smaller degree as compared to the other algorithms.

This can also be seen in Figure 6, whereby as the dimensions of the objective function increased the execution time of the ACSA increased, but by a smaller magnitude as compared to SCSA.

## 8. Conclusion

In this study, an aggressive cuckoo search algorithm (ACSA) is proposed and the algorithm is used to optimize uplink power in Code-Division Multiple Access based (CDMA) networks. In the ACSA, the concept of Eber Moll's model of a transistor is emulated to make the step size of the ACSA to be adaptive, and thus vary non-linearly from a smaller step size to a larger one. Likewise, the value of the probability of discovery is made to vary linearly from a larger probability value to a smaller set value and is dependent on the number of iterations of the ACSA. Lastly, Lévy flight in the cuckoo search algorithm was replaced with the Beta distribution. The ACSA was tested against 10 other standard optimization algorithms and 4 other advanced optimization algorithms in 23 benchmark optimization functions. The ACSA managed to outperform or obtain the same optimum value as the standard optimization algorithms in 18 of the 23 functions and outperformed or obtained the same optimum value as the other advanced optimization algorithms in 13 of the 23 functions. Moreover, the ACSA was used to determine the optimum uplink power in a CDMA-based network where each Mobile Station (MS) has a predetermined SINR value that it is supposed to meet. The ACSA was able to determine the power needed by all the MS optimally compared to the other algorithms in all three testing scenarios. The ACSA outperformed the other standard optimization algorithms in all three scenarios and outperformed the other advanced optimization algorithms in the third scenario.

## Figures and Tables

**Algorithm 1:**
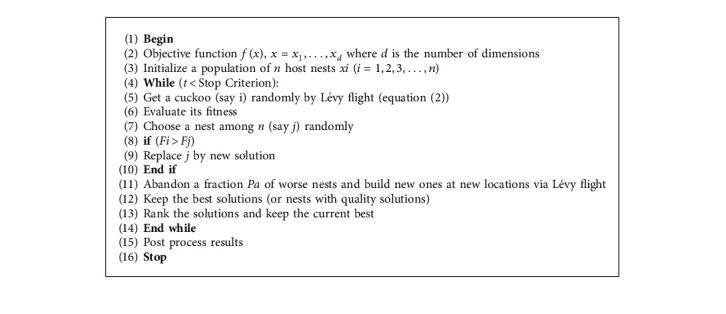
Pseudo Code of Standard Cuckoo Search Algorithm.

**Algorithm 2:**
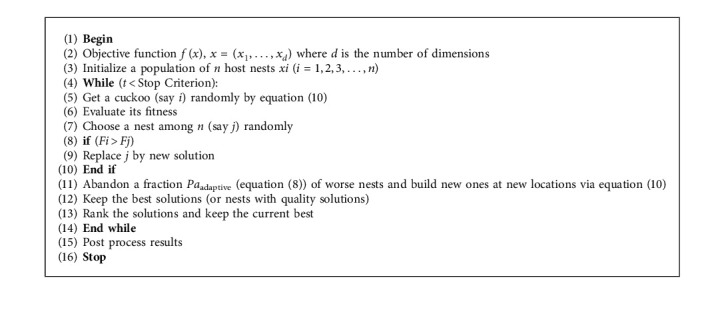
Pseudo Code of Aggressive Cuckoo Search Algorithm.

**Table 1:** Unimodal benchmark functions.

Function	Dimensions	Range	Optimum value
*F*1(*x*)=∑_*i*=1_^*n*^*x*_*i*_^2^	15	[−100, 100]	0
*F*2(*x*)=∑_*i*=1_^*n*^|*x*_*i*_|+∏_*i*=1_^*n*^|*x*_*i*_|	15	[−10, 10]	0
*F*3(*x*)=∑_*i*=1_^*n*^(∑_*j*−1_^*i*^*x*_*j*_)^2^	15	[−100, 100]	0
*F*4(*x*)=max_*i*_{|*x*_*i*_|, 1 ≤ *i* ≤ *n*}	15	[−100, 100]	0
*F*5(*x*)=∑_*i*=1_^*n*−1^[100(*x*_*i*+1_ − *x*_*i*_^2^)^2^+(*x*_*i*_+1)^2^]	15	[−30, 30]	0
*F*6(*x*)=∑_*i*=1_^*n*−1^([*x*_*i*_+0.5]^2^)	15	[−100, 100]	0
*F*7(*x*)=∑_*i*=1_^*n*−1^*ix*_*i*_^4^+random[0,1)	15	[−1.28, 1.28]	0

**Table 2:** Multimodal benchmark functions.

Function	Dimensions	Range	Optimum value
$F8\left(x\right)=\sum_{i=1}^{n}-x_{i}\sin\left(\sqrt{\left|x_{i}\right|}\right)\;$	15	[−500, 500]	−2094.9145
*F*9(*x*)=∑_*i*=1_^*n*^[*x*_*i*_^2^ − 10cos(2*πx*_*i*_)+10]	15	[−5.12, 5.12]	0
$F10\left(x\right)=-20\exp\left(-0.2\sqrt{\left(1/n\right)\sum_{i=1}^{n}x_{i}^{2}}\right)-\text{exp}\left(\left(1/n\right)\sum_{i=1}^{n}\cos\left(2\pi x_{i}\right)\right)+20+e$	15	[−32, 32]	0
$F11\left(x\right)=\left(1/4000\right)\sum_{i=1}^{n}x_{i}^{2}-\prod_{i=1}^{n}\cos\left(x_{i}/\sqrt{i}\right)+1$	15	[−600, 600]	0
*F*12(*x*)=(*π*/*n*){10sin(*πy*_*i*_)+ ∑_*i*=1_^*n*−1^(*y*_*i*_ − 1)^2^ [1+10sin^2^(*πy*_*i*+1_)]+(*y*_*n*_ − 1)^2^}+∑_*i*=1_^*n*^*u*(*x*_*i*_, 10,100,4)			
$y_{i}=1+\left(\left(x_{i}+1\right)/4\right)u\left(x_{i},a,k,m\right)=\left\{\begin{array}{c} k{\left(x_{i}-a\right)}^{m}x_{i}>a\\ 0-a<x_{i}<a\\ k{\left(-x_{i}-a\right)}^{m}x_{i}<-a \end{array}\right.$	15	[−50, 50]	0
*F*13(*x*)=0.1{sin^2^(3*πx*_1_)+∑_*i*=1_^*n*^(*x*_*i*_ − 1)^2^[1+ sin^2^(3*πx*_*i*_+1)]+ (*x*_*n*_ − 1)^2^[1+sin^2^(2*πx*_*n*_)]}+∑_*i*=1_^*n*^*u*(*x*_*i*_, 5,100,4)	15	[−50, 50]	0

**Table 3:** Fixed-dimension multimodal benchmark functions.

Function	Dimensions	Range	Optimum value
*F*14(*x*)=((1/500)+∑_*j*=1_^25^(1/*j*+∑_*i*=1_^2^(*x*_*i*_ − *a*_*ij*_)^6^))^−1^	2	[−65.536, 65.536]	1
*F*15(*x*)=∑_*i*=1_^11^*a*_*ij*_ − (*x*_1_(*b*_*i*_^2^+*b*_*i*_*x*_2_)/*b*_*i*_^2^+*b*_*i*_*x*_3_+*x*_4_)^2^	4	[−5, 5]	0.00030
*F*16(*x*)=4*x*_1_^2^ − 2.1*x*_1_^4^+(1/3)*x*_1_^6^+*x*_1_*x*_2_ − 4*x*_2_^2^+4*x*_2_^4^	2	[−5, 5]	−1.0316
*F*17(*x*)=(*x*_2_ − (5.1/4*π*^2^)*x*_1_^2^+(5/*π*)*x*_1_ − 6)^2^+10(1 − (1/8*π*))cos*x*_1_+10	2	*Lb* = [−5, 0], *Ub* = [10, 15]	0.398
*F*18(*x*)=[1+(*x*_1_+*x*_2_+1)^2^(19 − 14*x*_1_+3*x*_1_^2^ − 14*x*_2_+6*x*_1_*x*_2_+3*x*_2_^2^)] × [30+(2*x*_1_ − 3*x*_2_)^2^ × (18 − 32*x*_1_+12*x*_1_^2^+48*x*_2_ − 36*x*_1_*x*_2_+27*x*_2_^2^)]	2	[−2, 2]	3
*F*19(*x*)=−∑_*i*=1_^4^*c*_*i*_exp(−∑_*j*=1_^3^*a*_*ij*_(*x*_*j*_ − *p*_*ij*_)^2^)	3	[0, 1]	−3.86
*F*20(*x*)=−∑_*i*=1_^4^*c*_*i*_exp(−∑_*j*=1_^6^*a*_*ij*_(*x*_*j*_ − *p*_*ij*_)^2^)	6	[0, 1]	−3.32
*F*21(*x*)=−∑_*i*=1_^5^[(*X* − *a*_*i*_)(*X* − *a*_*i*_)^*T*^+*C*_*i*_]^−1^	4	[0, 10]	−10.1532
*F*22(*x*)=−∑_*i*=1_^7^[(*X* − *a*_*i*_)(*X* − *a*_*i*_)^*T*^+*C*_*i*_]^−1^	4	[0, 10]	−10.4028
*F*23(*x*)=−∑_*i*=1_^10^[(*X* − *a*_*i*_)(*X* − *a*_*i*_)^*T*^+*C*_*i*_]^−1^	4	[0, 10]	−10.5363

**Table 4:** Optimization results for ablation experiment.

Function		Cuckoo search algorithm variant	
		With increasing step size	With decreasing step size
*F*1	Mean	9.1727* E − *08	9.9413* E − *08
	Std. deviation	4.6146* E − *08	4.3413* E − *08

*F*2	Mean	5.5501* E − *05	3.0945* E − *05
	Std. deviation	1.6182* E − *05	1.0957* E − *05

*F*3	Mean	8.7521* E + *00	9.2051* E + *00
	Std. deviation	3.2950* E + *00	3.2103* E + *00

*F*4	Mean	6.0963* E − *01	6.4898* E − *01
	Std. deviation	8.7436* E − *02	1.0937* E − *01

*F*5	Mean	1.0882* E + *01	1.1213* E + *01
	Std. deviation	1.7445* E + *00	1.4061* E + *00

*F*6	Mean	9.1427* E − *08	1.0865* E − *07
	Std. deviation	3.6420* E − *08	5.1097* E − *08

*F*7	Mean	1.3694* E − *02	1.3638* E − *02
	Std. deviation	6.1983* E − *03	3.3336* E − *03

*F*8	Mean	−4.5028* E + *03	−4.5898* E + *03
	Std. deviation	2.6909* E + *02	1.7075* E + *02

*F*9	Mean	3.6834* E + *01	3.1332* E + *01
	Std. deviation	8.5542* E + *00	8.2324* E + *00

*F*10	Mean	3.7197* E − *03	9.6193* E − *03
	Std. deviation	2.7807* E − *03	6.5831* E − *03

*F*11	Mean	4.7514* E − *02	3.9768* E − *02
	Std. deviation	4.9314* E − *02	3.5430* E − *02

*F*12	Mean	1.7433* E − *05	2.4992* E − *05
	Std. deviation	3.3108* E − *05	5.4847* E − *05

*F*13	Mean	1.8434* E − *06	3.5573* E − *06
	Std. deviation	1.2647* E − *06	3.0531* E − *06

*F*14	Mean	9.9800* E − *01	9.9800* E − *01
	Std. deviation	2.2984* E − *16	2.2984* E − *16

*F*15	Mean	3.3698* E − *04	3.2268* E − *04
	Std. deviation	4.1346* E − *05	2.1580* E − *05

*F*16	Mean	−1.0316* E + *00	−1.0316* E + *00
	Std. deviation	4.5968* E − *16	4.5968* E − *16

*F*17	Mean	3.9789* E − *01	3.9789* E − *01
	Std. deviation	5.7460* E − *17	5.7460* E − *17

*F*18	Mean	3.0000* E + *00	3.0000* E + *00
	Std. deviation	0.0000* E + *00	0.0000* E + *00

*F*19	Mean	−3.8628* E + *00	−3.8628* E + *00
	Std. deviation	0.0000* E + *00	0.0000* E + *00

*F*20	Mean	−3.3220* E + *00	−3.3220* E + *00
	Std. deviation	9.1935* E − *16	9.1935* E − *16

*F*21	Mean	−1.0153* E + *01	−1.0153* E + *01
	Std. deviation	0.0000* E + *00	0.0000* E + *00

*F*22	Mean	−1.0403* E + *01	−1.0403* E + *01
	Std. deviation	0.0000* E + *00	0.0000* E + *00

*F*23	Mean	−1.0536* E + *01	−1.0536* E + *01
	Std. deviation	0.0000* E + *00	0.0000* E + *00

**Table 5:** Comparison of optimization results for the benchmark functions.

Function		SCSA	ACSA	ABC	BA	DE	GA	PSO	IWO	CA	FPA	FA
*F*1	Mean	3.3818* E − *02	4.8221* E − *48	8.7210* E − *07	1.2320* E + *04	5.8534* E − *12	4.9265* E − *02	4.6924* E − *21	3.4054* E + *03	2.8971* E − *06	3.5249* E + *00	6.9724* E − *04
	Std	1.0489* E − *02	1.8541* E − *47	4.5539* E − *07	3.9976* E + *03	2.8498* E − *12	2.5935* E − *02	1.5827* E − *20	2.1024* E + *03	1.0518* E − *05	6.8069* E − *01	2.3158* E − *04

*F*2	Mean	3.6316* E − *01	5.4215* E − *31	9.5988* E − *06	4.3246* E + *01	6.4768* E − *08	6.4478* E − *02	5.0236* E − *03	9.3886* E − *03	1.2374* E − *04	4.1165* E + *00	4.4977* E − *02
	Std	8.3076* E − *02	1.3225* E − *30	6.4962* E − *06	2.5286* E + *01	2.1020* E − *08	1.9402* E − *02	1.3176* E − *02	1.5097* E − *03	2.7733* E − *04	4.0906* E − *01	4.1924* E − *02

*F*3	Mean	3.6096* E + *01	3.4492* E − *16	2.4563* E + *03	1.1387* E + *04	1.7065* E + *03	9.0272* E + *00	3.8201* E − *08	6.8918* E + *03	4.1169* E + *02	2.9445* E + *00	2.6289* E + *01
	Std	1.0396* E + *01	1.3223* E − *15	5.5669* E + *02	3.0689* E + *03	2.9959* E + *02	6.0267* E + *00	5.4635* E − *08	3.4229* E + *03	4.3558* E + *02	1.0212* E + *00	2.2566* E + *01

*F*4	Mean	3.5917* E + *00	4.0529* E − *13	8.2312* E + *00	5.3939* E + *01	2.9403* E − *01	2.5320* E − *01	5.9640* E − *05	3.5661* E + *01	1.0181* E + *01	3.9812* E + *00	2.7999* E − *02
	Std	7.3019* E − *01	9.3867* E − *13	1.1115* E + *00	5.8552* E + *00	8.4086* E − *02	7.7749* E − *02	1.9293* E − *04	8.0374* E + *00	4.4153* E + *00	1.1863* E + *00	1.0959* E − *02

*F*5	Mean	3.0877* E + *01	9.7070* E + *00	6.4866* E + *01	1.0098* E + *04	2.8851* E + *01	3.9840* E + *01	7.9823* E + *00	2.5533* E + *01	1.4340* E + *02	9.1950* E + *00	6.7086* E + *01
	Std	4.4131* E + *00	2.7433* E − *01	4.9199* E + *01	2.0096* E + *04	1.4768* E + *01	4.1328* E + *01	1.5672* E + *00	2.8777* E + *01	1.7739* E + *02	1.6240* E + *00	5.6272* E + *01

*F*6	Mean	3.2855* E − *02	1.7559* E − *04	1.9348* E − *06	1.2338* E + *04	8.0607* E − *12	3.7144* E − *02	4.1693* E − *23	3.0308* E + *03	5.4237* E − *05	3.9806* E + *00	6.3867* E − *04
	Std	1.5169* E − *02	7.3491* E − *05	2.1138* E − *06	2.6851* E + *03	4.7690* E − *12	1.5452* E − *02	9.2542* E − *23	1.3492* E + *03	1.9120* E − *04	1.2075* E + *00	2.6802* E − *04

*F*7	Mean	2.2274* E − *02	1.8982* E − *03	1.9117* E − *02	2.3991* E − *02	1.3067* E − *02	4.2087* E − *03	1.8781* E − *02	5.9146* E − *03	4.7814* E − *02	9.8957* E − *01	2.5692* E − *02
	Std	8.7090* E − *03	1.5623* E − *03	7.8994* E − *03	1.4852* E − *02	3.9197* E − *03	2.4887* E − *03	8.3970* E − *03	2.5779* E − *03	2.0559* E − *02	1.6387* E − *01	1.7738* E − *02

*F*8	Mean	−4.6486* E + *03	−3.8558* E + *03	−1.0913* E + *61	−2.4868* E + *03	−6.2847* E + *03	−5.9339* E + *03	−3.0400* E + *46	−3.2591* E + *03	−4.2062* E + *14	−1.6365* E + *03	−3.2736* E + *03
	Std	1.6012* E + *02	1.5969* E + *02	2.8579* E + *61	4.0681* E + *02	1.8828* E − *12	1.6791* E + *02	1.1774* E + *47	4.0819* E + *02	1.6291* E + *15	2.3682* E + *02	4.8914* E + *02

*F*9	Mean	3.1695* E + *01	0.0000* E + *00	6.7237* E + *01	9.4719* E + *01	8.1612* E + *00	5.6675* E + *00	1.0878* E + *01	2.6401* E + *01	3.8009* E + *01	2.9790* E + *00	1.1476* E + *01
	Std	6.2335* E + *00	0.0000* E + *00	6.8697* E + *00	1.7623* E + *01	2.4959* E + *00	1.7744* E + *00	4.6129* E + *00	5.6265* E + *00	1.3743* E + *01	2.1954* E − *01	4.4635* E + *00

*F*10	Mean	3.9341* E + *00	8.8818* E − *16	8.2990* E − *03	1.9516* E + *01	1.0592* E − *06	9.3265* E − *02	1.1311* E − *12	1.6152* E + *01	8.8984* E − *01	9.0090* E + *00	9.0277* E − *03
	Std	9.3389* E − *01	2.0414* E − *31	3.6630* E − *03	7.6135* E − *01	2.6371* E − *07	3.0647* E − *02	2.0205* E − *12	6.5783* E + *00	7.0408* E − *01	1.5120* E + *00	2.1121* E − *03

*F*11	Mean	2.4302* E − *01	0.0000* E + *00	3.4224* E − *01	2.0881* E + *02	1.0428* E − *05	2.1564* E − *01	0.0000* E + *00	2.1296* E + *02	4.7890* E − *02	3.0669* E + *00	2.0263* E − *03
	Std	4.9034* E − *02	0.0000* E + *00	1.0019* E − *01	3.9837* E + *01	3.3906* E − *05	8.0892* E − *02	0.0000* E + *00	3.7163* E + *01	3.1015* E − *02	1.5805* E + *00	7.6404* E − *04

*F*12	Mean	8.2166* E − *01	7.6020* E − *04	8.4136* E − *03	5.3758* E + *04	3.5049* E − *13	2.4097* E − *04	3.7926* E − *25	2.3064* E + *01	2.6072* E + *00	4.7715* E + *00	1.8656* E − *05
	Std	3.6375* E − *01	4.8806* E − *04	1.6004* E − *02	6.7924* E + *04	1.9689* E − *13	1.1637* E − *04	9.4933* E − *25	8.0891* E + *00	2.4061* E + *00	8.7874* E − *01	1.5411* E − *05

*F*13	Mean	9.3809* E − *02	2.3459* E − *03	5.2368* E − *03	3.0639* E + *06	1.9593* E − *12	5.6940* E − *03	1.0886* E − *02	3.3461* E + *00	2.9212* E + *00	4.7208* E + *00	1.5266* E − *04
	Std	2.5401* E − *02	1.2862* E − *03	9.7319* E − *03	4.6657* E + *06	1.7209* E − *12	4.5369* E − *03	2.7474* E − *02	7.7382* E + *00	4.3843* E + *00	8.1817* E − *01	7.9179* E − *05

*F*14	Mean	9.9800* E − *01	9.9800* E − *01	9.9800* E − *01	7.9490* E + *00	9.9800* E − *01	9.9800* E − *01	1.2671* E + *01	1.1843* E + *01	1.8561* E + *00	7.4699* E − *04	1.6824* E + *00
	Std	2.2984* E − *16	2.2984* E − *16	9.1026* E − *06	5.3033* E + *00	2.2984* E − *16	2.2984* E − *16	3.6774* E − *15	5.8797* E + *00	1.3913* E + *00	8.3188* E − *04	7.4059* E − *01

*F*15	Mean	4.2591* E − *04	3.1446* E − *04	1.0949* E − *03	8.6098* E − *03	6.7353* E − *04	2.2167* E − *03	4.2038* E − *04	7.1031* E − *04	8.0023* E − *04	3.7440* E − *02	2.4773* E − *03
	Std	7.4102* E − *05	1.1029* E − *05	5.5643* E − *05	9.6919* E − *03	8.5902* E − *05	5.0268* E − *03	3.2962* E − *04	2.7133* E − *04	2.5479* E − *04	2.1783* E − *02	5.1404* E − *03

*F*16	Mean	−1.0316* E + *00	−1.0316* E + *00	−1.0316* E + *00	− 8.1396* E − *01	−1.0316* E + *00	−1.0316* E + *00	−1.0316* E + *00	−1.0316* E + *00	−1.0316* E + *00	−3.7475* E − *01	−1.0316* E + *00
	Std	9.3389* E − *01	2.0414* E − *31	3.6630* E − *03	7.6135* E − *01	2.6371* E − *07	3.0647* E − *02	2.0205* E − *12	6.5783* E + *00	7.0408* E − *01	1.5120* E + *00	2.1121* E − *03

*F*17	Mean	3.9789* E − *01	3.9789* E − *01	3.9789* E − *01	3.9789* E − *01	3.9789* E − *01	3.9789* E − *01	3.9789* E − *01	3.9789* E − *01	3.9789* E − *01	3.4750* E − *07	3.9789* E − *01
	Std	5.7460* E − *17	5.7460* E − *17	5.7460* E − *17	5.7460* E − *17	5.7460* E − *17	5.7460* E − *17	5.7460* E − *17	5.7460* E − *17	5.7460* E − *17	5.9170* E − *07	5.7460* E − *17

*F*18	Mean	3.0000* E + *00	3.0000* E + *00	3.0000* E + *00	8.4000* E + *00	3.0000* E + *00	3.0000* E + *00	4.8000* E + *00	3.0000* E + *00	3.0000* E + *00	2.0301* E − *06	3.0000* E + *00
	Std	0.0000* E + *00	0.0000* E + *00	0.0000* E + *00	1.1179* E + *01	0.0000* E + *00	0.0000* E + *00	6.9714* E + *00	0.0000* E + *00	0.0000* E + *00	1.2400* E − *06	0.0000* E + *00

*F*19	Mean	−3.8628* E + *00	−3.8628* E + *00	−3.8628* E + *00	−3.8628* E + *00	−3.8628* E + *00	−3.8628* E + *00	−3.8628* E + *00	−3.8628* E + *00	−3.8628* E + *00	−3.4963* E + *00	−3.8628* E + *00
	Std	0.0000* E + *00	0.0000* E + *00	0.0000* E + *00	0.0000* E + *00	0.0000* E + *00	0.0000* E + *00	0.0000* E + *00	0.0000* E + *00	0.0000* E + *00	2.7785* E − *01	0.0000* E + *00

*F*20	Mean	−3.3220* E + *00	−3.3220* E + *00	−3.3220* E + *00	−3.2507* E + *00	−3.3220* E + *00	−3.2586* E + *00	−3.2824* E + *00	−3.2031* E + *00	−3.2824* E + *00	−1.6511* E + *00	−3.2865* E + *00
	Std	9.1935* E − *16	9.1935* E − *16	3.5187* E − *05	6.0293* E − *02	9.1935* E − *16	6.1400* E − *02	5.8017* E − *02	2.5820* E − *05	5.8017* E − *02	4.5167* E − *01	6.1182* E − *02

*F*21	Mean	−1.0153* E + *01	−1.0153* E + *01	−1.0153* E + *01	−5.2831* E + *00	−9.9901* E + *00	−8.1540* E + *00	−5.0552* E + *00	−5.6430* E + *00	−6.9863* E + *00	−6.2422* E − *01	−9.3371* E + *00
	Std	0.0000* E + *00	0.0000* E + *00	0.0000* E + *00	2.7494* E + *00	6.3051* E − *01	3.4314* E + *00	0.0000* E + *00	3.4321* E + *00	3.5867* E + *00	3.0194* E − *01	2.2129* E + *00

*F*22	Mean	−1.0403* E + *01	−1.0403* E + *01	−1.0403* E + *01	−5.1089* E + *00	−1.0403* E + *01	−8.9376* E + *00	−5.0877* E + *00	−6.4827* E + *00	−9.5125* E + *00	−7.2652* E − *01	−1.0403* E + *01
	Std	0.0000* E + *00	0.0000* E + *00	0.0000* E + *00	2.9145* E + *00	2.5820* E − *04	3.0411* E + *00	9.1935* E − *16	3.8419* E + *00	2.3500* E + *00	3.2179* E − *01	0.0000* E + *00

*F*23	Mean	−1.0536* E + *01	−1.0536* E + *01	−1.0536* E + *01	−4.0544* E + *00	−1.0536* E + *01	−7.9558* E + *00	−5.1285* E + *00	−6.7777* E + *00	−7.6804* E + *00	−1.1969* E + *00	−1.0536* E + *01
	Std	0.0000* E + *00	0.0000* E + *00	0.0000* E + *00	2.7571* E + *00	0.0000* E + *00	3.7912* E + *00	9.1935* E − *16	3.7768* E + *00	3.7113* E + *00	6.0862* E − *01	0.0000* E + *00

**Table 6:** Comparison of optimization results from advanced algorithms.

Function		ACSA	GQPSO	IRGA	RACS	FSTDE
*F*1	Mean	4.8221* E − *48	1.9724* E − *91	1.4005* E − *19	7.6901* E − *16	1.2323* E − *07
	Std. deviation	1.8541* E − *47	4.1239* E − *91	5.4237* E − *19	5.6795* E − *16	4.5591* E − *08

*F*2	Mean	5.4215* E − *31	1.1509* E − *49	2.2875* E − *16	4.7214* E − *10	5.2382* E − *05
	Std. deviation	1.3225* E − *30	2.6290* E − *49	8.3263* E − *16	2.2922* E − *10	9.8743* E − *06

*F*3	Mean	3.4492* E − *16	3.1627* E − *46	2.8860* E + *01	1.1424* E + *01	3.3559* E + *03
	Std. deviation	1.3223* E − *15	1.2248* E − *45	3.0764* E + *01	1.0268* E + *01	7.2910* E + *02

*F*4	Mean	4.0529* E − *13	2.8701* E − *39	2.3161* E − *01	6.5397* E − *03	3.1662* E + *00
	Std. deviation	9.3867* E − *13	5.3079* E − *39	1.4892* E − *01	2.7908* E − *03	4.5167* E − *01

*F*5	Mean	9.7070* E + *00	1.2910* E + *01	1.7260* E + *01	8.9719* E + *00	5.4752* E + *01
	Std. deviation	2.7433* E − *01	9.9435* E − *02	2.1532* E + *01	1.5111* E + *00	2.4079* E + *01

*F*6	Mean	1.7559* E − *04	5.4841* E − *01	6.1296* E − *23	1.2358* E − *15	2.0152* E − *07
	Std. deviation	7.3491* E − *05	7.4729* E − *02	1.2339* E − *22	9.4064* E − *16	1.4882* E − *07

*F*7	Mean	1.8982* E − *03	1.0292* E − *04	3.0213* E − *03	8.5470* E − *03	2.6284* E − *02
	Std. deviation	1.5623* E − *03	9.4909* E − *05	1.5828* E − *03	2.9045* E − *03	8.2054* E − *03

*F*8	Mean	−3.8558* E + *03	−1.9422* E + *03	−6.1742* E + *03	−6.2847* E + *03	−2.8379* E + *76
	Std. deviation	1.5969* E + *02	1.2939* E + *02	1.0465* E + *02	1.8828* E − *12	4.9659* E + *76

*F*9	Mean	0.0000* E + *00	0.0000* E + *00	3.8218* E − *12	2.6954* E − *08	6.9869* E − *02
	Std. deviation	0.0000* E + *00	0.0000* E + *00	1.0837* E − *11	4.3614* E − *08	9.5101* E − *02

*F*10	Mean	8.8818* E − *16	8.8818* E − *16	2.3465* E − *11	1.0676* E − *08	1.8699* E − *04
	Std. deviation	2.0414* E − *31	2.0414* E − *31	3.6666* E − *11	3.5527* E − *09	4.6148* E − *05

*F*11	Mean	0.0000* E + *00	0.0000* E + *00	1.4426* E − *02	1.8368* E − *02	8.8549* E − *03
	Std. deviation	0.0000* E + *00	0.0000* E + *00	1.6758* E − *02	6.6206* E − *03	6.6708* E − *03

*F*12	Mean	7.6020* E − *04	7.1707* E − *02	1.2601* E − *20	9.3048* E − *16	5.3876* E − *09
	Std. deviation	4.8806* E − *04	1.2912* E − *02	4.5205* E − *20	7.5322* E − *16	2.6303* E − *09

*F*13	Mean	2.3459* E − *03	3.6423* E − *01	6.6567* E − *20	2.2345* E − *15	2.3757* E − *08
	Std. deviation	1.2862* E − *03	5.3472* E − *02	2.2354* E − *19	2.0989* E − *15	1.1320* E − *08

*F*14	Mean	9.9800* E − *01	2.7620* E + *00	9.9800* E − *01	9.9800* E − *01	9.9800* E − *01
	Std. deviation	2.2984* E − *16	2.2657* E + *00	2.2984* E − *16	2.2984* E − *16	2.2984* E − *16

*F*15	Mean	3.1446* E − *04	3.6353* E − *04	2.0484* E − *03	3.0749* E − *04	1.0605* E − *03
	Std. deviation	1.1029* E − *05	4.1497* E − *05	5.0683* E − *03	0.0000* E + *00	2.1523* E − *04

*F*16	Mean	−1.0316* E + *00	−1.0314* E + *00	−1.0316* E + *00	−1.0316* E + *00	−1.0316* E + *00
	Std. deviation	4.5968* E − *16	1.4075* E − *04	4.5968* E − *16	4.5968* E − *16	4.5968* E − *16

*F*17	Mean	3.9789* E − *01	4.0041* E − *01	3.9789* E − *01	3.9789* E − *01	3.9789* E − *01
	Std. deviation	5.7460* E − *17	3.5059* E − *03	5.7460* E − *17	5.7460* E − *17	5.7460* E − *17

*F*18	Mean	3.0000* E + *00	3.0001* E + *00	3.0000* E + *00	3.0000* E + *00	3.0000* E + *00
	Std. deviation	0.0000* E + *00	1.7915* E − *04	0.0000* E + *00	0.0000* E + *00	0.0000* E + *00

*F*19	Mean	−3.8628* E + *00	−3.8558* E + *00	−3.8628* E + *00	−3.8628* E + *00	−3.8628* E + *00
	Std. deviation	0.0000* E + *00	4.5270* E − *03	0.0000* E + *00	0.0000* E + *00	0.0000* E + *00

*F*20	Mean	−3.3220* E + *00	−3.0228* E + *00	−3.2903* E + *00	−3.3220* E + *00	−3.3204* E + *00
	Std. deviation	9.1935* E − *16	6.3832* E − *02	5.4425* E − *02	9.1935* E − *16	4.4347* E − *03

*F*21	Mean	−1.0153* E + *01	−4.4861* E + *00	−1.0153* E + *01	−1.0153* E + *01	−1.0152* E + *01
	Std. deviation	0.0000* E + *00	2.1697* E − *01	0.0000* E + *00	0.0000* E + *00	3.3594* E − *03

*F*22	Mean	−1.0403* E + *01	−4.6669* E + *00	−9.4486* E + *00	−1.0403* E + *01	−1.0403* E + *01
	Std. deviation	0.0000* E + *00	2.0743* E − *01	2.5251* E + *00	0.0000* E + *00	0.0000* E + *00

*F*23	Mean	−1.0536* E + *01	−4.5974* E + *00	−1.0089* E + *01	−1.0536* E + *01	−1.0536* E + *01
	Std. deviation	0.0000* E + *00	1.8971* E − *01	1.7301* E + *00	0.0000* E + *00	0.0000* E + *00

**Table 7:** Comparison of ACSA against the 10 other optimization algorithms on runtime in seconds.

Function	Timing in seconds										
	SCSA	ACS	ABC	BA	DE	GA	PSO	IWO	CA	FPA	FA
*F*1	0.23350	0.19976	0.83612	0.14544	0.52964	0.13972	0.03839	0.35092	2.23420	0.13210	0.78708
*F*2	0.24539	0.20572	0.86824	0.14434	0.54550	0.14442	0.04209	0.32486	2.27600	0.13283	0.80137
*F*3	0.49218	0.44686	1.11720	0.27144	0.64493	0.28165	0.16578	0.50632	2.52310	0.13392	0.92855
*F*4	0.23989	0.20061	0.85224	0.14951	0.52736	0.14571	0.03993	0.34404	2.41320	0.13305	0.81448
*F*5	0.29579	0.26231	0.93798	0.18561	0.57212	0.17226	0.06669	0.40304	2.40120	0.13263	0.83263
*F*6	0.23378	0.19766	0.85472	0.14524	0.54563	0.14325	0.03895	0.35519	2.36120	0.13185	0.80165
*F*7	0.36877	0.33887	1.01590	0.21414	0.58772	0.21021	0.10451	0.49662	2.43820	0.13382	0.87378
*F*8	0.28490	0.25478	1.04950	0.19612	0.57992	0.17219	0.05976	0.39868	2.35710	0.13623	0.83169
*F*9	0.25869	0.22004	0.89568	0.15365	0.54479	0.15849	0.04794	0.35004	2.36850	0.13384	0.81912
*F*10	0.27957	0.23557	0.94785	0.17987	0.58514	0.16651	0.04807	0.33403	2.36530	0.13726	0.84347
*F*11	0.31408	0.27288	1.00910	0.20132	0.62486	0.18819	0.07231	0.44287	2.41550	0.13403	0.84499
*F*12	0.66107	0.62268	1.37900	0.37513	0.80876	0.36280	0.25126	0.63189	2.50200	0.13173	0.99674
*F*13	0.68999	0.65314	1.45660	0.39948	0.84302	0.38126	0.26280	0.67952	2.70640	0.13381	1.02870
*F*14	1.48210	1.49200	2.33320	0.82751	1.21690	0.80241	0.68303	2.04150	1.10570	0.10988	1.35500
*F*15	0.19909	0.19723	0.87645	0.15704	0.52369	0.13857	0.04340	0.49802	0.76498	0.11581	0.73431
*F*16	0.18284	0.19618	0.87759	0.13167	0.47554	0.12953	0.03608	0.61993	0.37355	0.10845	0.71024
*F*17	0.17853	0.18176	0.85572	0.12801	0.50837	0.12708	0.03073	0.62991	0.41328	0.11968	0.76187
*F*18	0.39712	0.43054	2.28870	0.31962	1.28050	0.31055	0.06731	1.62700	0.99864	0.28960	1.81540
*F*19	0.20557	0.20650	0.90854	0.14340	0.51140	0.14572	0.04468	0.53982	0.54281	0.10881	0.71253
*F*20	0.22471	0.21872	0.90675	0.15602	0.54593	0.15004	0.04826	0.41306	0.99210	0.11398	0.74711
*F*21	0.48337	0.46856	1.22390	0.29008	0.66938	0.29144	0.17722	0.66183	0.88404	0.11234	0.86309
*F*22	0.58358	0.58199	1.34000	0.34576	0.71697	0.33000	0.23188	0.70044	0.92874	0.11234	0.90753
*F*23	0.78849	0.80828	1.60290	0.44727	0.85724	0.45297	0.33318	0.87908	1.04030	0.11827	1.04710

**Table 8:** Statistical analysis on evaluation metrics.

Test function	Wilcoxon's rank sum test	Aggressive cuckoo search algorithm (ACSA)									
		SCSA vs ACSA	ABC vs ACSA	BA vs ACSA	DE vs ACSA	GA vs ACSA	PSO vs ACSA	IWO vs ACSA	CA vs ACSA	FPA vs ACSA	FA vs ACSA
*F*1	*p* value	3.39180* E* − 06	3.39180* E* − 06	3.39180* E* − 06	3.39180* E* − 06	3.39180* E* − 06	3.39180* E* − 06	3.39180* E* − 06	3.39180* E* − 06	3.39180* E* − 06	3.39180* E* − 06
	*z* value	4.64550* E* + 00	4.64550* E* + 00	4.64550* E* + 00	4.64550* E* + 00	4.64550* E* + 00	4.64550* E* + 00	4.64550* E* + 00	4.64550* E* + 00	4.64550* E* + 00	4.64550* E* + 00
	*h* value	1	1	1	1	1	1	1	1	1	1

*F*2	*p* value	3.39180* E* − 06	3.39180* E* − 06	3.39180* E* − 06	3.39180* E* − 06	3.39180* E* − 06	3.39180* E* − 06	3.39180* E* − 06	3.39180* E* − 06	3.39180* E* − 06	3.39180* E* − 06
	*z* value	4.6455	4.6455	4.6455	4.6455	4.6455	4.6455	4.6455	4.6455	4.6455	4.6455
	*h* value	1	1	1	1	1	1	1	1	1	1

*F*3	*p* value	3.39180* E* − 06	3.39180* E* − 06	3.39180* E* − 06	3.39180* E* − 06	3.39180* E* − 06	3.39180* E* − 06	3.39180* E* − 06	3.39180* E* − 06	3.39180* E* − 06	3.39180* E* − 06
	*z* value	4.6455	4.6455	4.6455	4.6455	4.6455	4.6455	4.6455	4.6455	4.6455	4.6455
	*h* value	1	1	1	1	1	1	1	1	1	1

*F*4	*p* value	3.39180* E* − 06	3.39180* E* − 06	3.39180* E* − 06	3.39180* E* − 06	3.39180* E* − 06	3.39180* E* − 06	3.39180* E* − 06	3.39180* E* − 06	3.39180* E* − 06	3.39180* E* − 06
	*z* value	4.6455	4.6455	4.6455	4.6455	4.6455	4.6455	4.6455	4.6455	4.6455	4.6455
	*h* value	1	1	1	1	1	1	1	1	1	1

*F*5	*p* value	3.39180* E* − 06	3.39180* E* − 06	3.39180* E* − 06	3.39180* E* − 06	4.22470* E* − 04	2.46260* E* − 03	9.05850* E* − 04	5.73710* E* − 05	3.10170* E* − 02	3.39180* E* − 06
	*z* value	4.6455	4.6455	4.6455	4.6455	3.5256	−3.0279	3.3182	4.0234	−2.1569	4.6455
	*h* value	1	1	1	1	1	1	1	1	1	1

*F*6	*p* value	3.39180* E* − 06	3.39180* E* − 06	3.39180* E* − 06	3.39180* E* − 06	3.39180* E* − 06	3.39180* E* − 06	3.39180* E* − 06	3.38330* E* − 06	3.39180* E* − 06	1.60530* E* − 05
	*z* value	4.6455	−4.6455	4.6455	−4.6455	4.6455	−4.6455	4.6455	−4.6461	4.6455	4.3137
	*h* value	1	1	1	1	1	1	1	1	1	1

*F*7	*p* value	3.39180* E* − 06	3.39180* E* − 06	3.39180* E* − 06	3.39180* E* − 06	1.28220* E* − 02	3.39180* E* − 06	1.60530* E* − 05	3.39180* E* − 06	3.39180* E* − 06	5.05270* E* − 06
	*z* value	4.6455	4.6455	4.6455	4.6455	2.4887	4.6455	4.3137	4.6455	4.6455	4.5626
	*h* value	1	1	1	1	1	1	1	1	1	1

*F*8	*p* value	4.14320* E* − 06	3.39180* E* − 06	3.39180* E* − 06	3.39180* E* − 06	3.39180* E* − 06	4.50270* E* − 05	1.32950* E* − 05	3.39180* E* − 06	3.39180* E* − 06	1.60530* E* − 05
	*z* value	−4.6041	−4.6455	4.6455	−4.6455	−4.6455	4.08	4.3552	−4.6455	4.6455	4.3137
	*h* value	1	1	1	1	1	1	1	1	1	1

*F*9	*p* value	6.86620* E* − 07	6.86620* E* − 07	6.86620* E* − 07	6.86620* E* − 07	6.86620* E* − 07	6.86620* E* − 07	6.86620* E* − 07	6.86620* E* − 07	6.86620* E* − 07	6.86620* E* − 07
	*z* value	4.9651	4.9651	4.9651	4.9651	4.9651	4.9651	4.9651	4.9651	4.9651	4.9651
	*h* value	1	1	1	1	1	1	1	1	1	1

*F*10	*p* value	6.8662* E* − 07	6.8662* E* − 07	6.8662* E* − 07	6.8662* E* − 07	6.8662* E* − 07	6.7774* E* − 07	6.8662* E* − 07	6.8439* E* − 07	6.8662* E* − 07	6.8662* E* − 07
	*z* value	4.9651	4.9651	4.9651	4.9651	4.9651	4.9676	4.9651	4.9657	4.9651	4.9651
	*h* value	1	1	1	1	1	1	1	1	1	1

*F*11	*p* value	6.8662* E* − 07	6.8662* E* − 07	6.8662* E* − 07	6.8662* E* − 07	6.8662* E* − 07	1.0000* E* + 00	6.8662* E* − 07	6.8662* E* − 07	6.8662* E* − 07	6.8662* E* − 07
	*z* value	4.9651	4.9651	4.9651	4.9651	4.9651	0	4.9651	4.9651	4.9651	4.9651
	*h* value	1	1	1	1	1	0	1	1	1	1

*F*12	*p* value	3.3918* E* − 06	3.2301* E* − 03	3.3918* E* − 06	3.3918* E* − 06	2.6217* E* − 04	3.3918* E* − 06	3.3918* E* − 06	3.6906* E* − 03	3.3918* E* − 06	3.3918* E* − 06
	*z* value	4.6455	2.9449	4.6455	−4.6455	−3.6501	−4.6455	4.6455	2.9035	4.6455	−4.6455
	*h* value	1	1	1	1	1	1	1	1	1	1

*F*13	*p* value	3.3918* E* − 06	4.0679* E* − 01	3.3918* E* − 06	3.3918* E* − 06	7.9403* E* − 03	3.0942* E* − 02	3.6150* E* − 01	5.4521* E* − 03	3.3918* E* − 06	3.3918* E* − 06
	*z* value	4.6455	−0.82956	4.6455	−4.6455	2.6546	−2.1578	−0.91252	2.779	4.6455	−4.6455
	*h* value	1	0	1	1	1	1	0	1	1	1

*F*14	*p* value	0.0795850	0.0000027	0.0000027	0.0000005	0.0003331	0.0000024	0.0000027	0.3239100	0.0000027	0.0000027
	*z* value	1.7531	4.6938	4.6938	−5.0242	−3.5881	4.7158	4.6938	−0.98646	−4.6938	4.6938
	*h* value	0	1	1	1	1	1	1	0	1	1

*F*15	*p* value	1.3295* E* − 05	3.3918* E* − 06	3.3918* E* − 06	3.3918* E* − 06	2.7983* E* − 05	5.4521* E* − 03	1.0992* E* − 05	6.1516* E* − 06	3.3918* E* − 06	3.3918* E* − 06
	*z* value	4.3552	4.6455	4.6455	4.6455	4.1893	−2.779	4.3967	4.5211	4.6455	4.6455
	*h* value	1	1	1	1	1	1	1	1	1	1

*F*16	*p* value	0.0020889	2.27* E* − 06	0.088595	4.07* E* − 07	4.07* E* − 07	4.07* E* − 07	2.27* E* − 06	1.6885* E* − 04	2.27* E* − 06	2.27* E* − 06
	*z* value	−3.0773	4.7278	−1.7029	−5.0659	−5.0659	−5.0659	4.7278	2.9035	4.7278	4.7278
	*h* value	1	1	0	1	1	1	1	1	1	1

*F*17	*p* value	1.8145* E* − 05	3.3833* E* − 06	6.8439* E* − 07	6.8439* E* − 07	6.8439* E* − 07	6.8439* E* − 07	3.3833* E* − 06	6.8439* E* − 07	3.3833* E* − 06	3.3833* E* − 06
	*z* value	−4.2866	4.6461	−4.9657	−4.9657	−4.9657	−4.9657	4.6461	−4.9657	−4.6461	4.6461
	*h* value	1	1	1	1	1	1	1	1	1	1

*F*18	*p* value	7.6091* E* − 05	9.5756* E* − 07	9.3996* E* − 07	1.0000* E* + 00	9.4264* E* − 02	5.0142* E* − 04	9.5756* E* − 07	6.5372* E* − 03	9.5756* E* − 07	9.5756* E* − 07
	*z* value	3.9564	4.9002	4.9038	0	1.6733	3.48	4.9002	2.7195	−4.9002	4.9002
	*h* value	1	1	1	0	0	1	1	1	1	1

*F*19	*p* value	1.6414* E* − 01	1.2128* E* − 06	3.8553* E* − 07	2.7990* E* − 06	2.7990* E* − 06	2.7990* E* − 06	1.2567* E* − 06	1.2119* E* − 03	1.2567* E* − 06	1.2567* E* − 06
	*z* value	1.3913	4.8535	5.076	−4.685	−4.685	−4.685	4.8465	−3.2361	4.8465	4.8465
	*h* value	0	1	1	1	1	1	1	1	1	1

*F*20	*p* value	1.0000* E* + 00	4.2247* E* − 04	7.7153* E* − 01	1.1377* E* − 04	7.7155* E* − 01	7.6801* E* − 01	3.3918* E* − 06	3.5389* E* − 01	3.3918* E* − 06	5.1239* E* − 02
	*z* value	0	−3.5256	0.29038	−3.8592	−0.29035	−0.29498	4.6455	0.92707	4.6455	−1.9495
	*h* value	0	1	0	1	0	0	1	0	1	0

*F*21	*p* value	4.0200* E* − 05	5.7371* E* − 05	3.0961* E* − 02	5.6283* E* − 02	1.2469* E* − 01	1.2567* E* − 06	3.3918* E* − 06	7.7018* E* − 01	3.3918* E* − 06	3.3918* E* − 06
	*z* value	4.1063	−4.0234	2.1576	1.9088	−1.5354	4.8465	4.6455	0.29214	4.6455	4.6455
	*h* value	1	1	1	0	0	1	1	0	1	1

*F*22	*p* value	9.6615* E* − 05	3.3833* E* − 06	3.0979* E* − 02	4.0235* E* − 01	1.2473* E* − 01	2.3628* E* − 06	3.3918* E* − 06	3.5838* E* − 01	3.3918* E* − 06	3.3918* E* − 06
	*z* value	3.8989	−4.6461	2.1573	−0.83742	−1.5352	4.7196	4.6455	−0.91846	4.6455	4.6455
	*h* value	1	1	1	0	0	1	1	0	1	1

*F*23	*p* value	1.0992* E* − 05	3.3580* E* − 06	6.6627* E* − 04	4.5636* E* − 02	3.6112* E* − 01	1.8500* E* − 06	3.3918* E* − 06	1.2356* E* − 01	3.3918* E* − 06	3.3918* E* − 06
	*z* value	4.3967	−4.6476	3.4031	−1.9987	−0.91323	4.7692	4.6455	1.54	4.6455	4.6455
	*h* value	1	1	1	1	0	1	1	0	1	1

**Table 9:** Returned results of ACSA and other standard optimization algorithms for scenario 1.

Algorithm	Returned value from algorithm	Total power (mW)	SINR values of each mobile station (dB)
SCSA	6.7180* e + *16	3.2340* e + *06	3.0857 6.5276 2.9503 3.7025 2.9860

ACSA	2.7813* e + *03	2.7813* e + *03	3.0279 3.0333 3.0051 3.0002 3.0097

PSO	1.6669* e + *12	1.8503* e + *03	2.5997 2.5997 2.5997 2.5997 2.5997

CA	4.9413* e + *05	4.9413* e + *05	3.4289 5.8598 3.4912 3.4306 3.3293

ABC	2.9245* e + *05	2.9245* e + *05	3.9532 5.3618 3.6490 3.3117 3.3786

DE	3.0886* e + *03	3.0886* e + *03	3.0529 3.2305 3.0223 3.1257 3.1036

BA	4.9943* e + *21	2.3474* e + *06	−3.0218 15.6949 −3.2819 −2.5987 −1.3870

FA	1.0762* e + *06	1.0762* e + *06	3.8657 3.2196 3.8842 4.6311 4.2080

GA	2.7891* e + *03	2.7891* e + *03	3.0048 3.0398 3.0173 3.0204 3.0069

IWO	2.2551* e + *22	2.4979* e + *06	−2.7827 17.5497 −9.6257 −2.7127 −4.0556

**Table 10:** Returned results of ACSA and other advanced optimization algorithms for scenario 1.

Algorithm	Returned value from algorithm	Total power (mW)	SINR values of each mobile station (dB)
ACSA	1.0859* e + *03	1.0859* e + *03	3.0006 3.0063 3.0279 3.0044 3.0219

RACS	1.0688* e + *03	1.0688* e + *03	3.0005 3.0000 3.0008 3.0001 3.0002

GQPSO	1.6930* e + *13	1.4850* e + *01	−8.0862 −8.0662 −9.1052 −8.9064 −8.8860

IRGA	1.0690* e + *03	1.0690* e + *03	3.0006 3.0010 3.0008 3.0005 3.0000

FSTDE	1.05023* e + *05	1.05023* e + *05	4.6007 4.2824 3.2933 4.1381 3.432

**Table 11:** Returned results of ACSA and other standard optimization algorithms for scenario 2.

Algorithm	Returned value from algorithm	Total power (mW)	SINR values of each receiving node (dB) SIN*R*_1_ SIN*R*_2_ SIN*R*_3_ SIN*R*_4_ SIN*R*_5_
SCSA	1.9578* e + *06	1.9578* e + *06	4.0782 3.1685 4.8501 3.9451 3.5019

ACSA	8.6615* e + *04	8.6615* e + *04	3.0007 3.0024 3.0004 3.0009 3.0000

PSO	1.5912* e + *13	2.0820* e + *03	−6.6329 −6.6330 −6.6328 −6.6330 −6.6330

CA	3.5512* e + *05	3.5512* e + *05	3.0366 4.1623 4.1751 4.1612 3.0110

ABC	2.4507* e + *05	2.4507* e + *05	4.0564 3.4052 3.6729 3.5795 3.3408

DE	1.2821* e + *05	1.2821* e + *05	3.2833 3.5701 3.3450 3.2315 3.0989

BA	7.7463* e + *16	1.6941* e + *06	−4.9341 3.2219 6.5298 5.4585 8.8142

FA	1.2546* e + *05	1.2546* e + *05	3.0132 3.0105 3.0093 3.0258 4.0500

GA	8.6984* e + *04	8.6984* e + *04	3.0101 3.0081 3.0023 3.0040 3.0003

IWO	1.1725* e + *17	2.1732* e + *06	5.6861 5.1089 2.7159 4.8911 −0.2595

**Table 12:** Returned results of ACSA and other advanced optimization algorithms for scenario 2.

Algorithm	Returned value from algorithm	Total power (mW)	SIN*R* values of each mobile station (dB)
ACSA	2.4981* E + *05	2.4981* E + *05	3.0003 3.0000 3.0002 3.0004 3.0001

RACS	2.4974* E + *05	2.4974* E + *05	3.0000 3.0000 3.0000 3.0000 3.0000

GQPSO	1.6702* E + *13	4.8085* E + *03	−7.2965 −8.1011 −7.4467 −7.3607 −8.1087

IRGA	2.4984* E + *05	2.4984* E + *05	3.0004 3.0001 3.0004 3.0003 3.0000

FSTDE	3.4141* E + *05	3.4141* E + *05	3.2197 3.0833 3.2967 3.1819 3.3998

**Table 13:** Returned results of ACSA and other standard optimization algorithms for scenario 3.

Algorithm	Returned value from algorithm	Total power (mW)	SIN*R* values of each receiving node (dB)
SCSA	6.8176* E + *13	0.0150	−56.3922 −63.3367 −70.2903 −63.3124 −64.4378 −67.9588

ACSA	1.4544* E + *13	2.2869* E + *06	2.7841 2.7841 2.7841 2.7841 2.7841 2.7841

PSO	1.7349* E + *13	6.5014* E + *03	−9.9581 −9.9581 −9.9581 −9.9581 −9.9581 −9.9581

CA	1.4570* E + *13	2.2387* E + *06	2.7793 2.7793 2.7793 2.7793 2.7793 2.7793

ABC	1.4634* E + *13	2.1304* E + *06	2.7679 2.7679 2.7679 2.7679 2.7679 2.7679

DE	2.2595* E + *13	253.8245	−23.8316 −23.8255 −23.8259 −23.8122 −23.8302 −23.8322

BA	4.4517* E + *15	2.4952* E + *06	1.5088 −0.4148 −0.5029 8.8117 2.7728 −0.0866

FA	1.5916* E + *13	1.1834* E + *05	−0.0744 −0.0693 −0.0739 −0.0734 −0.0686 −0.0696

GA	1.7151* E + *13	8.0167* E + *03	−9.0984 −9.1001 −9.0979 −9.1004 −9.1008 −9.0990

IWO	2.8979* E + *16	2.3928* E + *06	8.4637 4.3753 −1.5520 −8.8044 5.5631 −11.5173

**Table 14:** Returned results of ACSA and other advanced optimization algorithms for scenario 3.

Algorithm	Returned value from algorithm	Total power (mW)	SINR values of each receiving node (dB)
ACSA	1.4544* E + *13	2.2869* E + *06	2.7840 2.7841 2.7841 2.7841 2.7841 2.7841

RACS	1.4573* E + *13	2.2867* E + *06	2.7838 2.7840 2.7839 2.7846 2.7841 2.7838

GQPSO	1.7592* E + *13	9.5587* E + *03	−9.0734 −7.5709 −8.8931 −9.0629 −8.0456 −7.8165

IRGA	1.5372* E + *13	9.0019* E + *05	2.4571 2.4571 2.4571 2.4571 2.4571 2.4571

FSTDE	1.5464* E + *13	1.9699* E + *06	2.7471 2.7424 2.7517 2.7514 2.7563 2.7435

**Table 15:** Returned values and total power of ACSA and other standard optimization algorithms in Scenario 1 in three more test cases.

Algorithm	Test case					
	Test 1		Test 2		Test 3	
	Returned value	Total power (mW)	Returned value	Total power (mW)	Returned value	Total power (mW)
SCSA	4.38* E + *13	0.01	6.13* E + *17	8.57* E + *04	3.86* E + *13	0.0100
ACSA	1.71* E + *03	1.71* E + *03	331.2068	331.2068	605.2781	605.2781
PSO	1.71* E + *03	1.71* E + *03	1.68* E + *13	6.4845	605.5045	605.5045
CA	1.71* E + *03	1.71* E + *03	349.6878	349.6878	1.2943* E + *05	1.2943* E + *05
ABC	7.77* E + *04	7.77* E + *04	5.78* E + *04	5.78* E + *04	4.6975* E + *04	4.6975* E + *04
DE	1.71* E + *03	1.71* E + *03	331.2072	331.2072	3.8618* E + *13	0.0100
BA	1.63* E + *19	1.35* E + *06	4.69* E + *22	9.27* E + *05	3.3030* E + *21	1.9445* E + *06
FA	2.10* E + *05	2.10* E + *05	3.60* E + *13	0.01	3.8618* E + *13	0.0100
GA	1.71* E + *03	1.71* E + *03	331.2945	331.2945	605.4585	605.4585
IWO	3.53* E + *20	7.96* E + *05	1.45* E + *24	1.83* E + *06	1.2339* E + *23	4.2270* E + *05

**Table 16:** Returned values and total power of ACSA and other advanced optimization algorithms in scenario 1 in three more test cases.

Algorithm	Test case					
	Test 1		Test 2		Test 3	
	Returned value	Total power (mW)	Returned value	Total power (mW)	Returned value	Total power (mW)
ACSA	2.81* E + *03	2.81* E + *03	2.34* E + *03	2.34* E + *03	3.69* E + *03	3.69* E + *03
RACS	2.79* E + *03	2.79* E + *03	2.33* E + *03	2.33* E + *03	3.68* E + *03	3.68* E + *03
GQPSO	1.68* E + *13	4.54* E + *01	1.55* E + *13	8.87* E + *01	1.68* E + *13	6.08* E + *01
IRGA	2.79* E + *03	2.79* E + *03	2.40* E + *03	2.40* E + *03	3.76* E + *03	3.76* E + *03
FSTDE	3.48* E + *04	3.48* E + *04	1.01* E + *05	1.01* E + *05	1.28* E + *05	1.28* E + *05

**Table 17:** Returned values and total power of ACSA and other standard optimization algorithms in scenario 2 in three more test cases.

Algorithm	Test case					
	Test 1		Test 2		Test 3	
	Returned value	Total power (mW)	Returned value	Total power (mW)	Returned value	Total power (mW)
SCSA	6.1818* e + *13	0.0125	1.2694* e + *06	1.2694* e + *06	2.0641* e + *12	2.7487* e + *06
ACSA	1.4469* e + *05	1.4469* e + *05	1.4945* e + *05	1.4945* e + *05	1.1732* e + *05	1.1732* e + *05
PSO	1.4202* e + *13	2.2278* e + *04	1.5464* e + *13	4.8485* e + *03	1.6207* e + *13	2.2838* e + *03
CA	4.3364* e + *05	4.3364* e + *05	2.2806* e + *05	2.2806* e + *05	4.4728* e + *05	4.4728* e + *05
ABC	3.3436* e + *05	3.3436* e + *05	3.7870* e + *05	3.7870* e + *05	2.1981* e + *05	2.1981* e + *05
DE	2.4106* e + *13	1.4072* e + *06	1.5023* e + *05	1.5023* e + *05	1.0835* e + *06	1.0835* e + *06
BA	1.0507* e + *17	1.1152* e + *06	3.7041* e + *06	3.7041* e + *06	4.4641* e + *17	1.3226* e + *06
FA	1.0091* e + *13	3.0693* e + *04	1.5005* e + *05	1.5005* e + *05	6.3762* e + *12	4.4233* e + *04
GA	1.4476* e + *05	1.4476* e + *05	1.4946* e + *05	1.4946* e + *05	1.1737* e + *05	1.1737* e + *05
IWO	1.6808* e + *17	1.0580* e + *06	1.9468* e + *16	3.1805* e + *06	8.0124* e + *17	8.0124* e + *17

**Table 18:** Returned values and total power of ACSA and other advanced optimization algorithms in scenario 2 in three more test cases.

Algorithm	Test case					
	Test 1		Test 2		Test 3	
	Returned value	Total power (mW)	Returned value	Total power (mW)	Returned value	Total power (mW)
ACSA	3.3646* E + *05	3.3646* E + *05	1.3850* E + *05	1.3850* E + *05	3.9960* E + *05	3.9960* E + *05
RACS	3.3644* E + *05	3.3644* E + *05	1.3847* E + *05	1.3847* E + *05	3.9956* E + *05	3.9956* E + *05
GQPSO	1.5358* E + *13	2.9261* E + *04	1.6743* E + *13	2.2283* E + *03	1.3861* E + *13	4.5963* E + *04
IRGA	3.3647* E + *05	3.3647* E + *05	1.3850* E + *05	1.3850* E + *05	3.9981* E + *05	3.9981* E + *05
FSTDE	4.1758* E + *05	4.1758* E + *05	1.5767* E + *05	1.5767 * E + *05	4.7408* E + *05	4.7408* E + *05

**Table 19:** Returned values and total power of ACSA and other standard optimization algorithms in scenario 3 in three more test cases.

Algorithm	Test case					
	Test 1		Test 2		Test 3	
	Returned value	Total power (mW)	Returned value	Total power (mW)	Returned value	Total power (mW)
SCSA	7.0641* E + *13	0.0150	6.4006* E + *13	0.0150	1.2913* E + *14	2.2206* E + *05
ACSA	1.4984* E + *13	2.5303* E + *06	1.3264* E + *13	3.8763* E + *06	1.3999* E + *13	3.5652* E + *06
PSO	1.7137* E + *13	1.3273* E + *04	1.7035* E + *13	7.8628* E + *03	1.7242* E + *13	8.0351* E + *03
CA	1.5296* E + *13	1.6735* E + *06	1.3746* E + *13	3.1428* E + *06	1.5451* E + *13	8.5323* E + *05
ABC	1.5002* E + *13	2.4715* E + *06	1.4558* E + *13	1.9445* E + *06	1.4703* E + *13	2.2291* E + *06
DE	4.4162* E + *13	0.7668	1.6351* E + *13	1.4943* E + *06	6.4893* E + *13	0.0160
BA	1.0194* E + *16	2.1026* E + *06	2.5283* E + *15	6.5356* E + *05	4.0160* E + *15	2.9678* E + *06
FA	1.6153* E + *13	2.2931* E + *05	1.5635* E + *13	4.2343* E + *05	1.7334* E + *13	2.0477* E + *05
GA	1.5518* E + *13	1.0805* E + *06	1.4630* E + *13	1.8402* E + *06	1.6387* E + *13	2.6052* E + *04
IWO	1.9954* E + *16	2.4647* E + *06	4.0982* E + *15	3.0969* E + *06	1.8434* E + *17	2.5480* E + *06

**Table 20:** Returned values and total power of ACSA and other advanced optimization algorithms in scenario 3 in three more test cases.

Algorithm	Test case					
	Test 1		Test 2		Test 3	
	Returned value	Total power (mW)	Returned value	Total power (mW)	Returned value	Total power (mW)
ACSA	1.4992* E + *13	2.5021* E + *06	1.3281* E + *13	1.9941* E + *06	1.4323* E + *13	2.3531* E + *06
RACS	1.5049* E + *13	2.4750* E + *06	1.3357* E + *13	1.9842* E + *06	1.4381* E + *13	2.3187* E + *06
GQPSO	1.7544* E + *13	1.0467* E + *04	1.7695* E + *13	2.6353* E + *03	1.7608* E + *13	5.0555* E + *03
IRGA	1.5522* E + *13	1.0691* E + *06	1.4100* E + *13	1.3541* E + *06	1.5161* E + *13	1.1057* E + *06
FSTDE	1.6413* E + *13	3.6050* E + *05	1.6867* E + *13	1.0827* E + *06	1.6307* E + *13	1.6819* E + *06

## Data Availability

The standard algorithms for comparisons were sourced from Mathworks website repository of algorithms.
